# Metabolic Syndrome and Acute Respiratory Distress Syndrome in Hospitalized Patients With COVID-19

**DOI:** 10.1001/jamanetworkopen.2021.40568

**Published:** 2021-12-22

**Authors:** Joshua L. Denson, Aaron S. Gillet, Yuanhao Zu, Margo Brown, Thaidan Pham, Yilin Yoshida, Franck Mauvais-Jarvis, Ivor S. Douglas, Mathew Moore, Kevin Tea, Andrew Wetherbie, Rachael Stevens, John Lefante, Jeffrey G. Shaffer, Donna Lee Armaignac, Katherine A. Belden, Margit Kaufman, Smith F. Heavner, Valerie C. Danesh, Sreekanth R. Cheruku, Catherine A. St. Hill, Karen Boman, Neha Deo, Vikas Bansal, Vishakha K. Kumar, Allan J. Walkey, Rahul Kashyap

**Affiliations:** 1 Section of Pulmonary Diseases, Critical Care, and Environmental Medicine, Deming Department of Medicine, Tulane University School of Medicine, New Orleans, Louisiana; 2 Department of Biostatistics and Data Science, Tulane University School of Public Health and Tropical Medicine, New Orleans, Louisiana; 3 Section of Endocrinology and Metabolism, Deming Department of Medicine, Tulane University School of Medicine, New Orleans, Louisiana; 4 Southeast Louisiana Veterans Affairs Healthcare System, New Orleans; 5 Division of Pulmonary Sciences & Critical Care Medicine, Denver Health Medical Center, Denver, Colorado; 6 University of Colorado, Anschutz School of Medicine, Aurora; 7 Center for Advanced Analytics, Baptist Health South Florida, Miami; 8 Division of Infectious Diseases, Thomas Jefferson University Hospital, Philadelphia, Pennsylvania; 9 Englewood Health, Englewood, New Jersey; 10 Prisma Health Department of Medicine, Prisma Health Upstate, Greenville, South Carolina; 11 Baylor Scott & White Health, Department of Nursing, Dallas, Texas; 12 Divisions of Cardiothoracic Anesthesiology and Critical Care Medicine, Department of Anesthesiology and Pain Management, UT Southwestern Medical Center, Dallas, Texas; 13 Department of Care Delivery Research, Allina Health, Minneapolis, Minnesota; 14 Society of Critical Care Medicine, Mount Prospect, Illinois; 15 Department of Anesthesia and Perioperative Medicine, Mayo Clinic, Rochester, Minnesota; 16 The Pulmonary Center, Division of Pulmonary, Allergy, Sleep and Critical Care; Department of Medicine, Boston University School of Medicine, Boston, Massachusetts

## Abstract

**Importance:**

Obesity, diabetes, and hypertension are common comorbidities in patients with severe COVID-19, yet little is known about the risk of acute respiratory distress syndrome (ARDS) or death in patients with COVID-19 and metabolic syndrome.

**Objective:**

To determine whether metabolic syndrome is associated with an increased risk of ARDS and death from COVID-19.

**Design, Setting, and Participants:**

This multicenter cohort study used data from the Society of Critical Care Medicine Discovery Viral Respiratory Illness Universal Study collected from 181 hospitals across 26 countries from February 15, 2020, to February 18, 2021. Outcomes were compared between patients with metabolic syndrome (defined as ≥3 of the following criteria: obesity, prediabetes or diabetes, hypertension, and dyslipidemia) and a control population without metabolic syndrome. Participants included adult patients hospitalized for COVID-19 during the study period who had a completed discharge status. Data were analyzed from February 22 to October 5, 2021.

**Exposures:**

Exposures were SARS-CoV-2 infection, metabolic syndrome, obesity, prediabetes or diabetes, hypertension, and/or dyslipidemia.

**Main Outcomes and Measures:**

The primary outcome was in-hospital mortality. Secondary outcomes included ARDS, intensive care unit (ICU) admission, need for invasive mechanical ventilation, and length of stay (LOS).

**Results:**

Among 46 441 patients hospitalized with COVID-19, 29 040 patients (mean [SD] age, 61.2 [17.8] years; 13 059 [45.0%] women and 15713 [54.1%] men; 6797 Black patients [23.4%], 5325 Hispanic patients [18.3%], and 16 507 White patients [57.8%]) met inclusion criteria. A total of 5069 patients (17.5%) with metabolic syndrome were compared with 23 971 control patients (82.5%) without metabolic syndrome. In adjusted analyses, metabolic syndrome was associated with increased risk of ICU admission (adjusted odds ratio [aOR], 1.32 [95% CI, 1.14-1.53]), invasive mechanical ventilation (aOR, 1.45 [95% CI, 1.28-1.65]), ARDS (aOR, 1.36 [95% CI, 1.12-1.66]), and mortality (aOR, 1.19 [95% CI, 1.08-1.31]) and prolonged hospital LOS (median [IQR], 8.0 [4.2-15.8] days vs 6.8 [3.4-13.0] days; *P* < .001) and ICU LOS (median [IQR], 7.0 [2.8-15.0] days vs 6.4 [2.7-13.0] days; *P* < .001). Each additional metabolic syndrome criterion was associated with increased risk of ARDS in an additive fashion (1 criterion: 1147 patients with ARDS [10.4%]; *P* = .83; 2 criteria: 1191 patients with ARDS [15.3%]; *P* < .001; 3 criteria: 817 patients with ARDS [19.3%]; *P* < .001; 4 criteria: 203 patients with ARDS [24.3%]; *P* < .001).

**Conclusions and Relevance:**

These findings suggest that metabolic syndrome was associated with increased risks of ARDS and death in patients hospitalized with COVID-19. The association with ARDS was cumulative for each metabolic syndrome criteria present.

## Key Points

**Question:**

What is the risk of acute respiratory distress syndrome (ARDS) and death in patients with COVID-19 with metabolic syndrome?

**Findings:**

In this cohort study including 46 441 patients hospitalized for COVID-19, metabolic syndrome was associated with significantly increased odds of ARDS and death. With each metabolic syndrome criterion added from 1 to 4 criteria, the risk of ARDS significantly increased in an additive fashion.

**Meaning:**

These findings suggest that metabolic syndrome and its associated comorbidities were critical risk factors associated with COVID-19–related ARDS and death.

## Teaser

This cohort study examines the association of metabolic syndrome with risk of acute respiratory distress syndrome and death in hospitalized patients with COVID-19.

## Introduction

COVID-19 has affected more than 236 million people worldwide, causing more than 4.8 million deaths as of October 6, 2021.^1^ The US has been heavily encumbered with COVID-19, reporting more than 44 million infections and 707 000 deaths.^1^ The course of severe COVID-19 manifests with mild disease early on (<5-7 days), which progresses to pneumonia, acute hypoxic respiratory failure, and acute respiratory distress syndrome (ARDS) in a subset of patients after 8 to 15 days.^2^ This critically ill subset of patients experiences disproportionately prolonged hospitalizations (median, 17.1 days) and higher mortality (40.8%-71.6%),^3^ yet early identification of who will progress to critical illness remains uncertain.

Early studies suggested that obesity, diabetes, and hypertension^4,5,6^ were individually associated with increased COVID-19 severity, yet the mechanisms remain poorly understood. One possibility is that the clustering of obesity, prediabetes or diabetes, hypertension, and dyslipidemia, as a discrete phenotype, metabolic syndrome, is associated with high risk for severe disease.^7^ According to the Centers for Disease Control and Prevention, more than one-third (34.2%) of US adults meet the criteria for metabolic syndrome, with some regions having a metabolic syndrome prevalence of more than 40%.^8,9^ Metabolic syndrome is a chronic low-grade inflammatory state characterized by subtly elevated acute-phase reactant levels^10^ and endothelial dysfunction,^11^ which are also seen in COVID-19 and ARDS.^12,13,14^ Therefore, the combined characteristics of metabolic syndrome–associated comorbidities may be associated with driving COVID-19 mortality via systemic inflammation and endothelial dysfunction. In this study, we hypothesized that the additive or synergistic characteristics of metabolic syndrome–associated comorbidities are independently associated with increased risk for ARDS and mortality in patients with COVID-19.

## Methods

The data registry used in this cohort study was granted exempt status for human participant research by the institutional review board at Mayo Clinic. Each individual investigative site received local institutional review board approval and a data use agreement prior to the initiation of data collection. Per the study protocol approved at Mayo Clinic and each investigative site, informed consent was waived under Common Rule 45 CFR 46.116. This study is reported following the Strengthening the Reporting of Observational Studies in Epidemiology (STROBE) reporting guideline.

### Design, Setting, and Participants

This prospective cohort study used data from 181 hospitals across 26 countries from February 15, 2020, to February 18, 2021, using data collected as part of the Society of Critical Care Medicine Discovery Viral Respiratory Illness Universal Study (VIRUS) COVID-19 registry^15^ for patients hospitalized with COVID-19.^3^ VIRUS uses standard data collection procedures, which have been reported previously,^15^ to maximize the quality and fidelity of data elements. Data were entered in an electronic case report form using Research Electronic Data Capture.^16^

Inclusion criteria included hospitalized, adult (age ≥18 years) patients with COVID-19 confirmed by reverse transcription–polymerase chain reaction testing. Exclusion criteria excluded any patient without prior research authorization, any non–COVID-19–related admissions, any patient without a completed discharge status, any patient without documented age, and any patient entered from a hospital site with a case volume of fewer than 10 participants. Patients were retrospectively divided into 2 groups: those with metabolic syndrome and control patients, and outcomes of hospital discharge or death were evaluated. Patients with metabolic syndrome were identified from admission data using modified World Health Organization criteria^17^ as previously described^7^ and as having at least 3 of the following: prediabetes (hemoglobin A_1c_ ≥5.7% [to convert to proportion of total hemoglobin, multiply by 0.01]), history of diabetes, or diabetes medication use; obesity (body mass index [BMI; calculated as weight in kilograms divided by height in meters squared] ≥30); history of hypertension or antihypertensive medication use; and serum triglyceride (TG) level of 150 mg/dL or greater (to convert to millimoles per liter, multiply by 0.0113), serum high-density lipoprotein (HDL) level less than 50 mg/dL for women and less than 40 mg/dL for men (to convert to millimoles per liter, multiply by 0.0259), or cholesterol-lowering medication use with history of dyslipidemia. Since TG and HDL laboratory measurements are frequently unavailable during hospital admission, these 2 criteria were combined into a single dyslipidemia criterion^18^ to improve applicability in real-world practice and facilitate reproducibility by other investigators.^7,19^ The control cohort included any patient in the population not meeting the definition for metabolic syndrome at admission in the same study period as patients with metabolic syndrome.

Data elements for this study included demographics, medications, admission BMI, comorbid conditions to estimate the Elixhauser Comorbidity Index,^20^ worst level of oxygen support for each patient’s hospitalization, last recorded hemoglobin A_1c_ level, serum TG level, and serum HDL level. Serum TG and HDL levels were obtained from laboratory values most recent to the index hospitalization within 1 year prior to admission, when available, as these levels may change with acute inflammatory episodes.^21^ If measurements prior to hospitalization were not recorded, admission serum TG or HDL values were used when available; however, TG levels measured during or after administration of propofol were omitted, since propofol may lead to hypertriglyceridemia.^22^

### Outcomes

The primary outcome was hospital mortality. Secondary outcomes included a diagnosis of ARDS determined by site investigators using the Berlin Criteria,^23^ intensive care unit (ICU) admission, invasive mechanical ventilation, need for noninvasive ventilation, supplemental oxygen use, hospital and ICU length of stay (LOS), and the worst level of oxygen support needed during hospitalization, defined by the lowest score on a 5-category ordinal scale adapted from the World Health Organization scale,^24^ with 5 indicating death; 4, receiving invasive mechanical ventilation or extracorporeal membrane oxygenation (ECMO); 3, receiving noninvasive ventilation or high-flow oxygen devices; 2, requiring supplemental oxygen low flow (<15 L/min); and 1, not requiring supplemental oxygen. The primary outcome and secondary outcomes were compared between metabolic syndrome and control groups. Given the high prevalence of metabolic syndrome in the US, we compared rates of metabolic syndrome and each individual criterion (obesity, prediabetes or diabetes, hypertension, and dyslipidemia) in US and non-US hospital sites. Lastly, the primary outcome was then compared between patients hospitalized at US or non-US sites and stratified by metabolic syndrome.

Additionally, subgroup comparisons were performed to study the association of COVID-19 severity among patients with each metabolic syndrome criterion. First, to determine the risk increase associated with each cumulative metabolic syndrome criterion added, we defined subgroups according to the number of metabolic syndrome criteria present (ie, 0, 1, 2, 3, or 4 criteria) and compared mortality, ARDS, and LOS among them. The second subgroup analysis compared patients with each individual metabolic syndrome–associated criterion alone (eg, patients with obesity but without hypertension, dyslipidemia, or prediabetes or diabetes) with patients with no metabolic syndrome criteria (0 of 4 criteria) to explore which conditions, if any, may be associated with the greatest risk.

### Statistical Analysis

The main objective was to determine whether metabolic syndrome was associated with greater mortality compared with patients without metabolic syndrome. We used *t* test to compare means of numerical variables in different groups, and Mood median test was used for comparison of medians, with an α of 0.05 to determine statistical significance. Pearson χ^2^ test was used to compare the difference of distribution in groups for categorical variables. For comparison of the primary outcome and key secondary outcomes among metabolic syndrome and control groups, a clustered multivariable logistic regression model (or clustered multivariable linear regression model when appropriate) was constructed with the cluster of study site that patients came from and also including the following covariates selected using background knowledge of the factors connecting exposure to outcome: age, self-identified sex or gender (abstracted from health records and reported according to each site’s policy), self-identified race and ethnicity, hospital case volume (as a measure of hospital site variation), and Elixhauser Comorbidity Index.^20^ Quantile regression was used to test the difference of median of LOS, and a cumulative logistic model was used for the ordinal scale.

After inclusion and exclusion criteria, missing BMI data were substantial. Given that BMI is criterion standard to determine obesity and is one of the criteria to determine metabolic syndrome, incomplete data could result in possible misclassification bias. As such, additional steps were taken to account for this missingness. Since these data were assumed to be missing completely at random, a multiple imputation technique was used to create 20 imputed data sets to account for missing BMI values. Imputed data sets were analyzed separately and were reported as a pooled outcome across imputations. Multiple imputation and statistical analyses were performed using SAS Enterprise Guide version 6.1, and SAS, version 9.4 (SAS Institute). Figures were created using Prism version 9.0.1 (GraphPad). *P* values were 2-sided, and statistical significance was set at *P* = .05. Data were analyzed from February 22 to October 5, 2021.

## Results

### Study Population

**Figure 1.**
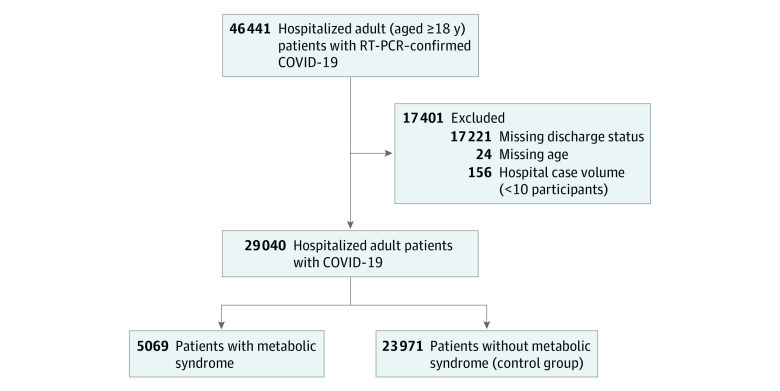
Cohort of Patients in Viral Respiratory Illness Universal Study Database Metabolic syndrome was defined as meeting at least 3 of the following criteria: prediabetes (hemoglobin A_1c_ ≥5.7%), diabetes, or diabetic medication use; obesity (body mass index [calculated as weight in kilograms divided by height in meters squared] ≥30); hypertension or antihypertensive medication use; and dyslipidemia.

Among 46 441 patients with COVID-19 who were hospitalized at 181 hospitals during the study period, 135 hospitals (74.6%) and 29 040 patients (62.5%) met eligibility criteria and were included in analyses (Figure 1). The mean (SD) age was 61.2 (17.8) years, and there were 13 059 (45.0%) women and 15 713 (54.1%) men. The full cohort included 6797 Black patients (23.4%), 5325 Hispanic patients (18.3%), and 16 507 White patients (57.8%) (Table 1). Baseline characteristics of eligible patients, divided into 5069 patients (17.5%) with metabolic syndrome and 23 971 control patients (82.5%), are presented in Table 1. The most common age category was 61 to 70 years (21.5%). The overall sample was predominantly hospitalized in the US (25 520 patients [87.9%]), with a mean (SD) BMI of 30.6 (6.7) and Elixhauser Comorbidity Score of 3.1 (6.6). The most common metabolic syndrome comorbid conditions were hypertension (15 581 patients [53.7%]), prediabetes or diabetes (9718 patients [33.5%]), and obesity (14 322 patients [49.3%]).

**Table 1.** Baseline Characteristics for Included Patients

Characteristics	Patients, No. (%)		
	Total (n = 29 040)	Metabolic syndrome (n = 5069)	Control (n = 23 971)
Age, y			
Mean (SD)	61.2 (17.8)	63.9 (13.3)	60.7 (18.5)
18-30	1726 (5.9)	35 (0.7)	1691 (7.18)
31-40	2400 (8.3)	205 (4.0)	2195 (9.2)
41-50	3720 (12.8)	561 (11.1)	3159 (13.2)
51-60	5522 (19.0)	1149 (22.7)	4373 (18.2)
61-70	6256 (21.5)	1484 (29.3)	4772 (19.9)
71-80	5169 (17.8)	1121 (22.1)	4048 (16.9)
>80	4247 (14.6)	514 (10.1)	3733 (15.6)
Sex or gender			
Men	15 713 (54.1)	2482 (49.0)	13 231 (55.2)
Women	13 059 (45.0)	2587 (51.0)	10 472 (43.7)
Nonbinary or unknown	268 (0.9)	0	268 (1.1)
Race			
American Indian or Alaska Native	180 (0.6)	37 (0.7)	143 (0.6)
Black	6797 (23.4)	1821 (35.9)	4976 (20.8)
Native Hawaiian or other Pacific Islander	51 (0.2)	8 (0.2)	43 (0.2)
White	16 507 (56.8)	2597 (51.2)	13 910 (58.0)
Asian	2386 (8.2)	183 (3.6)	2203 (9.2)
Other^a^	3119 (10.7)	423 (8.3)	2696 (11.2)
Hispanic ethnicity	5325 (18.3)	887 (17.5)	4438 (18.5)
Admitted at US hospital site	25 520 (87.9)	4785 (94.4)	20 735 (86.5)
BMI, mean (SD)	30.6 (6.7)	35.2 (7.3)	29.6 (6.1)
Elixhauser score, mean (SD)^b^	3.1 (6.6)	4.1 (7.4)	2.9 (6.4)
Metabolic syndrome comorbidities, No. (%)			
Prediabetes or diabetes^c^	9718 (33.5)	4658 (91.9)	5060 (21.1)
Obesity^d^	14 322 (49.3)	4447 (87.7)	9875 (41.2)
Hypertension^e^	15 581 (53.7)	4933 (97.3)	10 648 (44.4)
Dyslipidemia^f^	3029 (10.4)	2006 (39.6)	1023 (4.3)

Abbreviation: BMI, body mass index (calculated as weight in kilograms divided by height in meters squared).

^a^ Includes individuals who reported mixed, other, and unknown race.

^b^ Modified criteria as the Elixhauser scores for fluid or electrolyte disorders and alcohol abuse were unavailable, and diabetes with complications was combined with diabetes without complications.

^c^ Defined as hemoglobin A_1c_ level of 5.7% or higher, diabetes history, or diabetic medication use.

^d^ Defined as BMI of 30 or greater.

^e^ Defined as hypertension history or antihypertensive medication use.

^f^ Defined as high-density lipoprotein less than 50 mg/dL for women and less than 40 mg/dL for men (to convert to millimoles per liter, multiply by 0.0259), triglycerides level greater than 150 mg/dL (to convert to millimoles per liter, multiply by 0.0113), or cholesterol-lowering medication use with dyslipidemia history.

### Outcomes in Metabolic Syndrome vs Control Groups

Patients with metabolic syndrome, compared with control patients, were more likely to be women (2587 women [51.0%] vs 10 472 women [44.7%]), older (mean [SD] age, 63.9 [13.3] years vs 60.7 [18.5] years), and Black (1821 patients [35.9%] vs 4976 patients [20.8%]) (Table 1). In the primary analyses shown in Table 2, 1024 patients with metabolic syndrome (20.2%) died in the hospital, compared with 2828 control patients (16.0%) (unadjusted odds ratio [OR], 1.39 [95% CI, 1.23-1.58]). In multivariable analyses, metabolic syndrome remained significantly associated with in-hospital mortality (adjusted OR [aOR], 1.19 [95% CI, 1.08-1.31]). Patients with metabolic syndrome were also more likely to develop ARDS than control patients (1020 patients [20.1%] vs 2867 patients [12.0%]; aOR, 1.36 [95% CI, 1.12-1.66]). Similarly, other key secondary outcomes were significantly increased in patients with metabolic syndrome compared with control patients, including the need for ICU care (2451 patients [48.4%] vs 8594 patients [35.9%]; aOR, 1.32 [95% CI, 1.14-1.53]) and invasive mechanical ventilation (1428 patients [28.2%] vs 4084 patients [17.0%]; aOR, 1.45 [95% CI, 1.28-1.65]), as well as longer hospital LOS (median [IQR], 8.0 [4.2-15.8] days vs 6.8 [3.4-13.0] days; *P* < .001) and ICU LOS (median [IQR], 7.0 [2.8-15.0] days vs 6.4 [2.7-13.0] days; *P* < .001).

**Table 2.** Outcomes in Patients With Metabolic Syndrome vs Control Patients

Outcomes	Patients, No. (%)		OR (95% CI)	Adjusted OR (95% CI)^a^
	Metabolic syndrome	Control		
Hospital mortality	1024 (20.2)	2828 (16.0)	1.39 (1.23-1.58)^c^	1.19 (1.08-1.31)^c^
ARDS^b^	1020 (20.1)	2867 (12.0)	1.85 (1.46-2.34)^c^	1.36 (1.12-1.66)^c^
ICU	2451 (48.4)	8594 (35.9)	1.67 (1.36-2.05)^c^	1.32 (1.14-1.53)^c^
Invasive mechanical ventilation	1428 (28.2)	4084 (17.0)	1.87 (1.52-2.29)^c^	1.45 (1.28-1.65)^c^
Noninvasive mechanical ventilation	1444 (28.5)	3905 (16.3)	2.02 (1.63-2.50)^c^	1.64 (1.42-1.89)^c^
Supplemental oxygen	2289 (45.2)	7210 (30.1)	1.87 (1.44-2.44)^c^	1.45 (1.22-1.71)^c^
Length of stay, d				
Hospital				
Median (IQR)	8.0 (4.2-15.8)	6.8 (3.4-13.0)	0.78 (0.52-1.04)^c^^,^^d^	
Mean (95% CI)	12.4 (12.0-12.8)	10.5 (10.4-10.7)	1.18 (0.48-1.88)^c^^,^^d^	
ICU				
Median (IQR)	7.0 (2.8-15.0)	6.4 (2.7-13.0)	1.01 (0.57-1.45)^c^^,^^d^	
Mean (95% CI)	10.8 (10.4-11.3)	10.0 (9.7-10.3)	1.23 (0.61-1.85)^c^^,^^d^	

Abbreviations: ARDS, acute respiratory distress syndrome; ICU, intensive care unit; OR, odds ratio.

^a^ Calculated with clustered multivariable logistic regression model adjusted for age, sex, race, ethnicity, hospital case volume, and Elixhauser score.

^b^ ARDS was identified according to Berlin Criteria by site investigator.

^c^ *P* < .001.

^d^ Presented as adjusted differences calculated with clustered multivariable linear regression model adjusted for age, sex, race, ethnicity, hospital case volume, and Elixhauser score.

**Figure 2.**
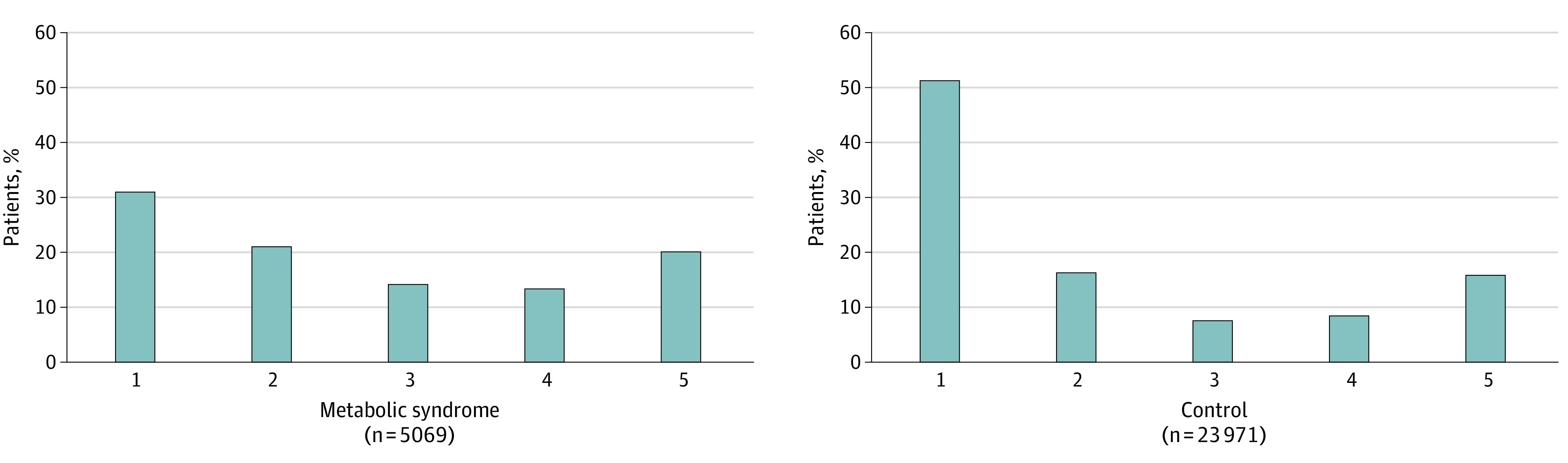
Worst 5-Category Ordinal Scale Scores Among Patients With Metabolic Syndrome vs Control Patients Score of 5 indicates death; 4, receiving invasive mechanical ventilation or extracorporeal membrane oxygenation; 3, receiving noninvasive ventilation or high-flow oxygen devices; 2, requiring supplemental oxygen; and 1, not requiring supplemental oxygen. A cumulative multivariable model demonstrated a significant increase in the odds that patients with metabolic syndrome would experience a 1-point worse ordinal scale score compared with control patients (crude odds ratio, 1.43 [95% CI, 1.35-1.52]).

Furthermore, patients with metabolic syndrome consistently required higher levels of oxygen support, as measured by the worst 5-category ordinal scale score during their hospitalization, including supplemental oxygen, noninvasive ventilation or high-flow oxygen devices, and invasive mechanical ventilation or ECMO compared with control patients (Figure 2). A cumulative multivariable model demonstrated a significant increase in the odds that patients with metabolic syndrome would experience a 1-point worse ordinal scale score compared with control patients (aOR, 1.43 [95% CI, 1.35-1.52]; *P* < .001).

### Additive Association of Metabolic Syndrome Criteria With Severe Outcomes

**Figure 3.**
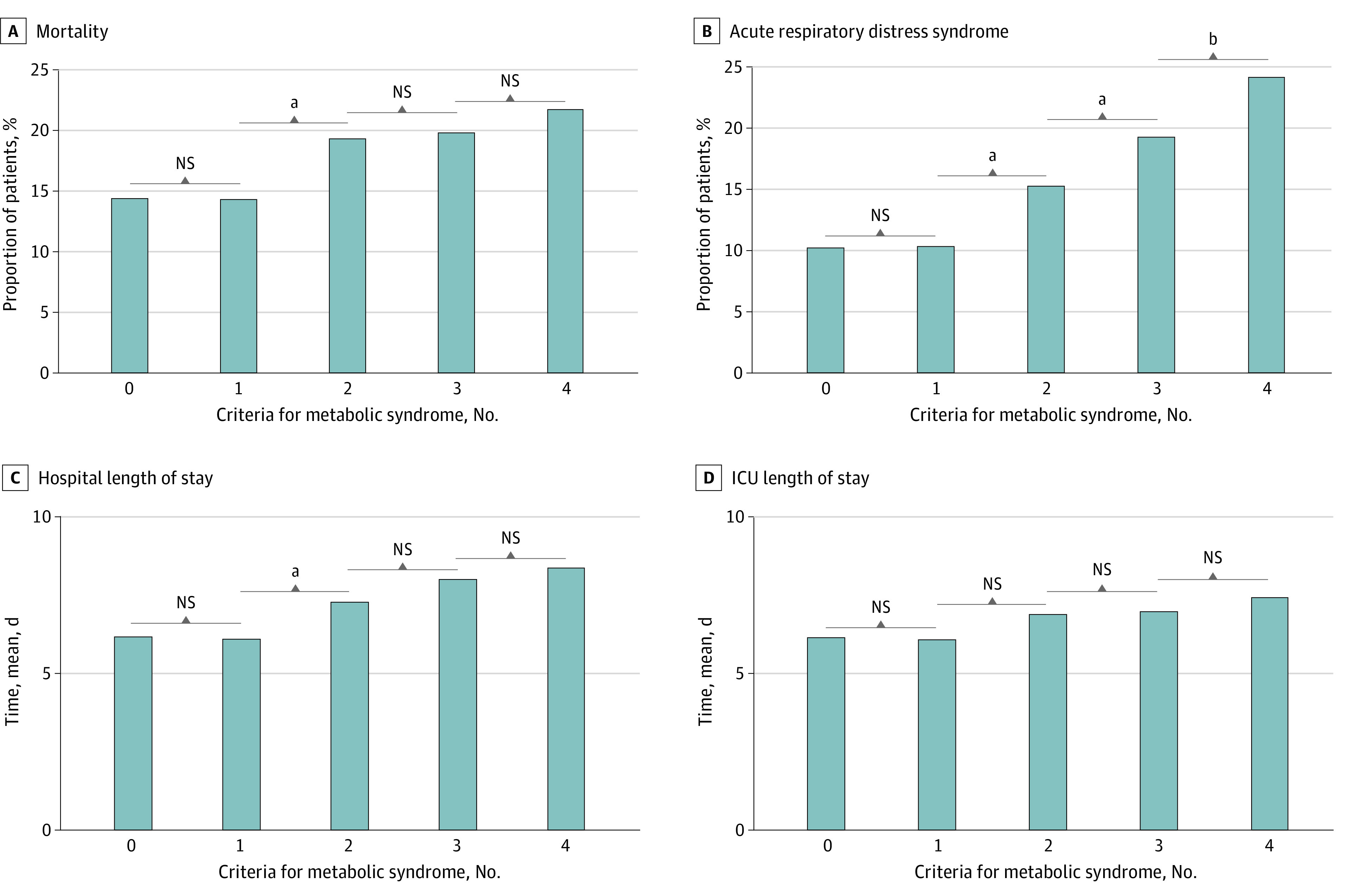
Additive Associations of Metabolic Syndrome Individual Criteria With Outcomes Of 4 metabolic syndrome criteria, 5148 patients had 0; 11 040 patients had 1; 7783 patients had 2; 4232 patients had 3; and 837 patients had 4. ICU indicates intensive care unit; NS, not significant. ^a^*P* < .001. ^b^*P* < .01.

The outcomes of patients with 0, 1, 2, 3, and 4 of 4 metabolic syndrome criteria are portrayed in Figure 3. With each metabolic syndrome criterion added from 1 to 2, 3, and 4 of 4 criteria, the proportion of patients who developed ARDS increased significantly (1 criterion: 1147 of 11040 patients [10.4%] with ARDS; *P* = .83; 2 criteria: 1191 of 7783 patients [15.3%] with ARDS; *P* < .001; 3 criteria: 817 of 4232 patients [19.3%] with ARDS; *P* < .001; 4 criteria: 203 of 837 patients [24.3%] with ARDS; *P* < .001). Similar findings were noted for hospital mortality and LOS when metabolic syndrome criterion were added from 1 to 2, but all other comparisons were not statistically significant.

### Metabolic Syndrome Individual Conditions

In additional subgroup analyses, patients with only 1 individual metabolic syndrome–associated comorbid condition were compared with a separate cohort of patients who did not have any of the metabolic syndrome–associated conditions (eFigure 1 in Supplement 1). Compared with patients without these metabolic syndrome risk factors, rates of ARDS were significantly increased for patients with prediabetes or diabetes, hypertension, and dyslipidemia, but the opposite association was seen with obesity. Hospital mortality was also significantly increased for patients with prediabetes or diabetes and hypertension, but not dyslipidemia, compared with patients without metabolic syndrome risk factors. Again, obesity was associated with a significant improvement in mortality (eFigure 1 in Supplement 1).

### Global and Hospital Case Volume Comparisons

Metabolic syndrome was significantly more common among patients with COVID-19 admitted to US hospitals compared with those admitted to non-US hospitals (4785 of 25 520 patients [18.8%] in US hospitals vs 284 of 3520 patients [8.1%] in non-US hospitals; *P* < .001). Furthermore, US hospitals, compared with non-US hospitals, admitted significantly higher proportions of patients with obesity (13 456 patients [52.7%] vs 866 patients [24.6%]; *P* < .001), prediabetes or diabetes (8602 patients [33.7%] vs 1116 patients [31.7%]; *P* = .02), hypertension (13 979 patients [54.8%] vs 1602 patients [45.5%]; *P* < .001), and dyslipidemia (2843 patients [11.1%] vs 186 patients [6.1%]; *P* < .001). However, hospital mortality did not differ between US and non-US hospitals in multivariable analyses (aOR, 0.64 [95% CI, 0.38-1.08]), with similar findings noted when patients were stratified by metabolic syndrome: in the metabolic syndrome group, 32 patients (19.5%) died in US hospitals vs 92 patients (32.4%) died in non-US hospitals (aOR, 0.69 [95% CI, 0.40-1.20]). These findings may be partly associated with increasing hospital case volumes, as shown in eFigure 2 in Supplement 1, which demonstrated a significant decline in hospital mortality associated with higher-volume hospitals (>1000 beds) compared with lower-volume hospitals (<50 beds) (aOR, 0.35 [95% CI, 0.24-0.51]; *P* < .001).

## Discussion

In this international, multicenter, prospective cohort study including 29 040 adults hospitalized with COVID-19, the presence of metabolic syndrome was associated with significantly increased odds of death and ARDS irrespective of age, sex, race, ethnicity, hospital case volume, and comorbid conditions. The 4% absolute mortality rate difference between patients with metabolic syndrome and control patients was prominent, with similarly high rates of ARDS and invasive mechanical ventilation in patients with metabolic syndrome compared with control patients. Additionally, the proportion of patients who developed ARDS was significantly increased in a stepwise and additive fashion with each metabolic syndrome criterion. Greater resource utilization with more frequent ICU admission and higher disease severity among patients with metabolic syndrome patients were also associated with prolonged hospital and ICU LOS compared with control patients.

Several studies have investigated the association between COVID-19 and individual metabolic diseases, such as obesity,^5^ diabetes,^25^ hypertension,^26^ and dyslipidemia^27^; yet how these different conditions are mechanistically associated with COVID-19 risk and illness severity remain to be fully elucidated.^28^ In smaller series, metabolic syndrome and its chronic low-grade inflammatory state^29^ have been postulated as instrumental in predisposing patients to ARDS and subsequently mortality.^7,19^ Our findings support this hypothesis, as patients with metabolic syndrome were not only at higher risk of ARDS and severe outcomes overall, but each additional metabolic syndrome criteria added was associated with greater risk of ARDS in an additive fashion among patients with 1, 2, 3, or 4 metabolic syndrome criteria.

Furthermore, although ARDS and death were increased among patients with metabolic syndrome, we also found that at every level of respiratory support, patients with metabolic syndrome experienced worse outcomes than control patients, with 43% increased risk of reaching a 1-point worse ordinal scale score. Specifically, metabolic syndrome patients experienced increased invasive mechanical ventilation or ECMO, increased noninvasive ventilation or high-flow oxygen support, and increased supplemental oxygen use compared with patients without metabolic syndrome. Measuring these different levels of respiratory support are valuable, as both ARDS and COVID-19 are heterogeneous disease states with varying levels of severity yet imperfect measurement characteristics.^30^ Moreover, there is growing evidence of specific subphenotypes in non–COVID-19 ARDS,^31^ and the use of respiratory support may be considered as a means to capture the spectrum of ARDS and respiratory failure.

The connection between metabolic disease and critical illness is well established. In fact, the so-called *obesity paradox*, a term given to the association of high BMI and increased susceptibility to more severe illness while paradoxically being associated with improved survival is well-described in ARDS.^32^ It remains unclear if the obesity paradox holds true in patients with COVID-19^33^ or if this finding can be attributed to collider bias, but our data, consistent with smaller studies published previously,^34,35^ may support this conclusion. Given the high rates of metabolic syndrome, obesity, and diabetes in the US,^8^ one hypothesis for why the US led the world in COVID-19 cases and deaths could be the high prevalence of metabolic syndrome in this population. Although our data show significantly increased rates of metabolic syndrome for patients in US hospitals compared with non-US hospitals, there were no significant differences in adjusted analyses comparing mortality between US and non-US sites. One explanation could be the association between hospital case volume and mortality, which demonstrated a significant decline when the higher-volume hospitals were compared with lower-volume hospitals, but these findings require further exploration. Nonetheless, these results highlight a potential reason why the COVID-19 pandemic may have disproportionately affected the US in terms of critical illness burden.

### Limitations

There are several important limitations that require consideration. Although VIRUS is one of the largest observational registries collecting data on patients with COVID-19, the conclusions drawn cannot imply causation. This study used multivariable regression analyses to address potential confounding, but unmeasured confounders, such as health care access, health system factors, or socioeconomic factors, may have contributed. Also, selection and information bias were considered important limitations owing to the unavailability of data from the electronic medical record. However, to mitigate this potential bias, several analyses were performed to support the statistical approach in addressing incomplete data. Additionally, complete patient phenotyping was not available, so concurrent bacterial infections and other concomitant causes of mortality could not be explored. Furthermore, we were not able to assess consistency of adherence with lung protective ventilation strategies, an established high-quality intervention associated with improved survival. This has been demonstrated to be challenging in patients who are obese and may have acted as an unmeasured confounder.

## Conclusions

This cohort study found that metabolic syndrome, diagnosed by the clustering of obesity, prediabetes or diabetes, hypertension, and dyslipidemia, was associated with significantly increased mortality and ARDS in a global population of hospitalized patients with COVID-19. This increased risk was cumulative, with the proportion of ARDS increasing with each added metabolic syndrome criteria.

## Notes

Supplement 1. **eFigure 1.** Associations of Individual Metabolic Syndrome Comorbidities With Outcomes **eFigure 2.** Association of Hospital Case Volume With Mortality

Supplement 2.
Group Information
