# Supplementary material for: Metabolic Syndrome and Acute Respiratory Distress Syndrome in Hospitalized Patients With COVID-19
Source: JAMA Netw Open. 2021 Dec 22;4(12):e2140568. doi: 10.1001/jamanetworkopen.2021.40568 (PMC8696573; doi:10.1001/jamanetworkopen.2021.40568)
Supplement: Supplement 1. — eFigure 1. Associations of Individual Metabolic Syndrome Comorbidities With Outcomes eFigure 2. Association of Hospital Case Volume With Mortality [file jamanetwopen-e2140568-s001.pdf]

Supplemental Online Content

Denson JL, Gillet AS, Zu Y, et al; Society of Critical Care Medicine Discovery Viral Infection and Respiratory Illness Universal Study (VIRUS): COVID-19 Registry Investigator Group. Metabolic syndrome and acute respiratory distress syndrome in hospitalized patients with COVID-19. *JAMA Netw Open*. 2021;4(12):e2140568. doi:10.1001/jamanetworkopen.2021.40568

eFigure 1. Associations of Individual Metabolic Syndrome Comorbidities With Outcomes
eFigure 2. Association of Hospital Case Volume With Mortality

This supplemental material has been provided by the authors to give readers additional information about their work.

© 2021 Denson JL et al. *JAMA Network Open.*

eFigure 1. Associations of Individual Metabolic Syndrome Comorbidities With Outcomes

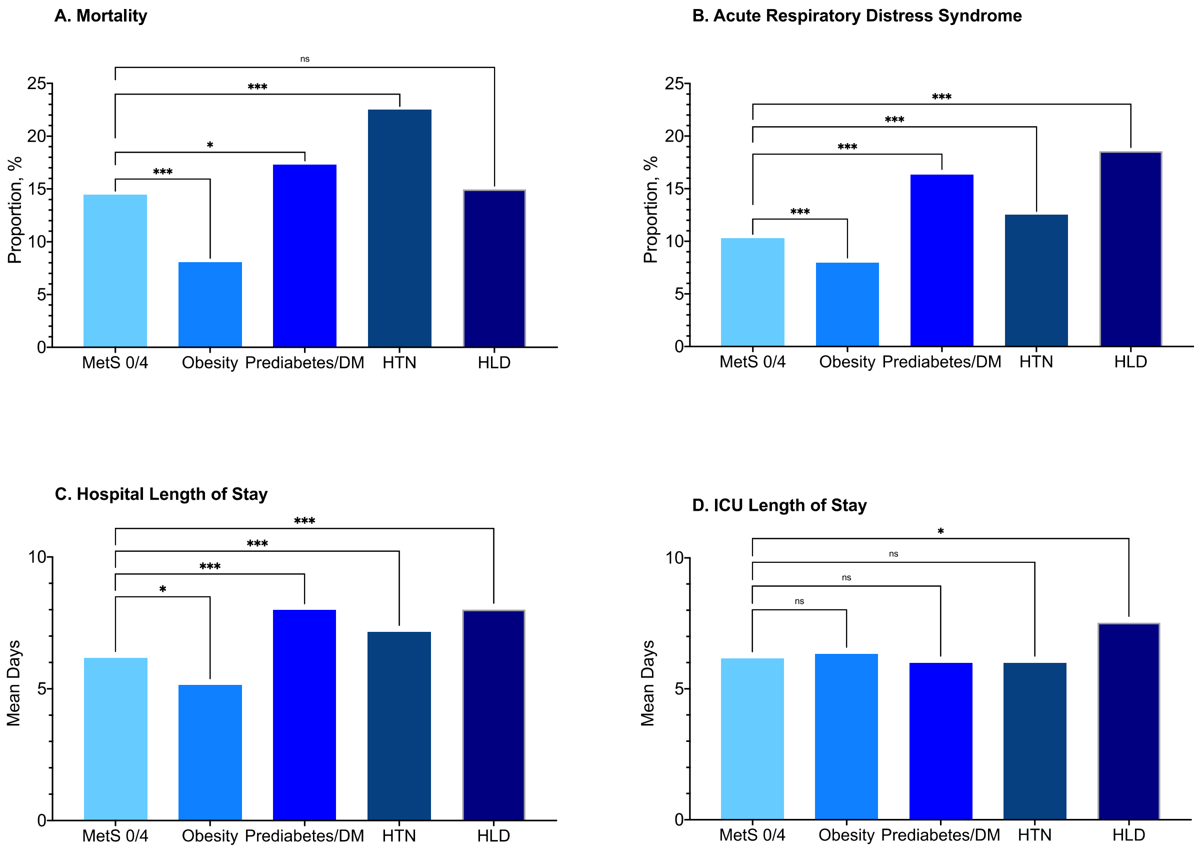

eFigure 1: Panels A and B: Bar graphs of proportion of hospital mortality and Acute Respiratory Distress Syndrome among MetS 0/4, obesity, prediabetes/DM, HTN and dyslipidemia. Panels C and D: Bar graphs of mean hospital and ICU length of stay in days for patients with obesity, prediabetes/DM, HTN and dyslipidemia compared to MetS 0/4. Each bar represents patients who were eligible for that individual MetS criteria without any other simultaneous MetS conditions present. MetS=Metabolic syndrome. DM=Diabetes mellitus. HTN=Hypertension. ICU=Intensive Care Unit. MetS 0/4 n=5,148, obesity n=5,744, prediabetes/DM n=1,005, HTN n=4,097, and dyslipidemia=194. *p<0.05. **p=<0.01.***p=<0.001.

© 2021 Denson JL et al. *JAMA Network Open.*

eFigure 2. Association of Hospital Case Volume With Mortality

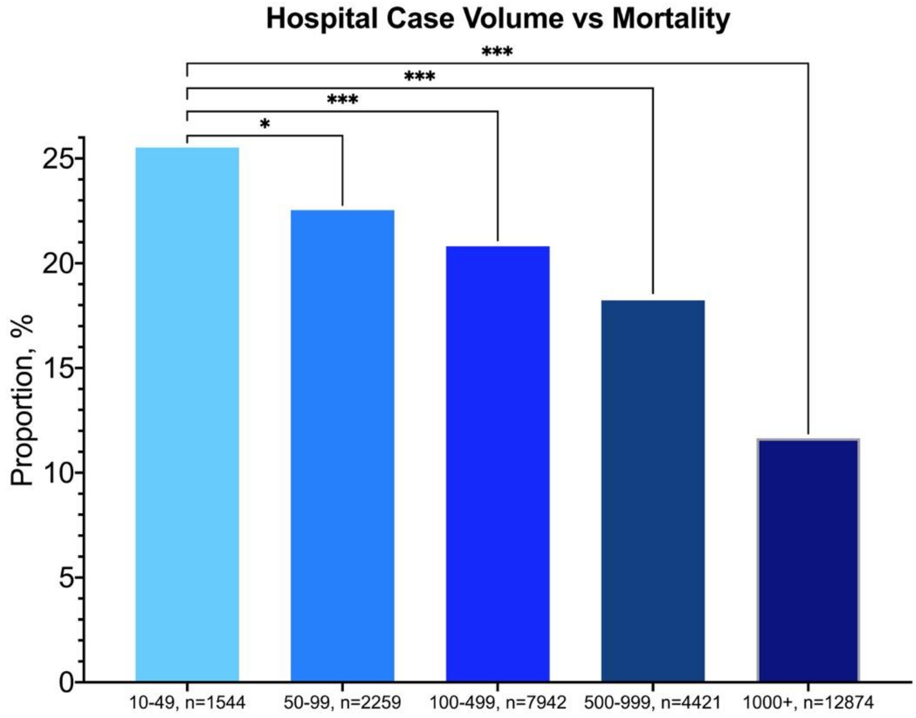

eFigure 2: Bar graph of hospital case volume vs mortality in proportions. *p<0.05. ***p=<0.001.

© 2021 Denson JL et al. *JAMA Network Open.*
