# Supplementary material for: Metabolic Syndrome and Acute Respiratory Distress Syndrome in Hospitalized Patients With COVID-19
Source: JAMA Netw Open. 2021 Dec 22;4(12):e2140568. doi: 10.1001/jamanetworkopen.2021.40568 (PMC8696573; doi:10.1001/jamanetworkopen.2021.40568)
Supplement: Supplement 2. — Group Information [file jamanetwopen-e2140568-s002.pdf]

Supplemental Online Content: Nonauthor Collaborators

*Indicates required information. Only first name, last name, and suffix will appear in PubMed.

***Group Name(s): Society of Critical Care Medicine Discovery Viral Infection and Respiratory Illness Universal Study (VIRUS): COVID-19 Registry [illegible]**

***First Name and Middle Initial(s)**	***Last Name**	***Suffix (eg, Jr, III)**	Academic Degrees	Institution	Location (city, state/province, country)	Role or Contribution, eg, chair, principal investigator	Group (if more than 1 Group listed in the byline) and/or Subgroup (eg, Steering Committee)
Jean-Baptiste	Mesland			Centre Hospitalier Jolimont	Belgium		
Pierre	Henin			Centre Hospitalier Jolimont	Belgium		
Hélène	Petre			Centre Hospitalier Jolimont	Belgium		
Isabelle	Buelens			Centre Hospitalier Jolimont	Belgium		
Anne-Catherine	Gerard			Centre Hospitalier Jolimont	Belgium		
Philippe	Clevenbergh			The Brugmann University Hospital	Belgium		
Rolando Claure	Del Granado			Clinica Los Olivos	Bolvia		
Jose A	Mercado			Clinica Los Olivos	Bolvia		
Esdenka	Vega-Terraza			Clinica Los Olivos	Bolvia		
Maria F	Iturricha-Caceres			Clinica Los Olivos	Bolvia		
Ruben	Garza			Mackenzie Health	Canada		
Eric	Chu			Mackenzie Health	Canada		
Victoria	Chan			Mackenzie Health	Canada		
Oscar Y	Gavidia			Clinica Medical SAS	Columbia		
Felipe	Pachon			Clinica Medical SAS	Columbia		
Mohamed El	Kassas			Helwan University	Egypt		
Ahmed	Tawheed			Helwan University	Egypt		
Estela	Pineda			CEMESA Hospital	Hondurus		
Gabina María	Reyes Guillen			Honduras Medical Center	Hondurus		
Helin Archaga	Soto			Honduras Medical Center	Hondurus		
Ana Karen	Vallecillo Lizardo			Honduras Medical Center	Hondurus		
Smitha S	Segu			Bangalore Medical College and Resea	India		
Tuhin	Chakraborty			Bangalore Medical College and Resea	India		
Epcebha	Joyce			Bangalore Medical College and Resea	India		
Phani Sreeharsha	Kasumalla			Bangalore Medical College and Resea	India		
Girish	Vadgaonkar			BSES MG Hospital	India		
Rekha	Ediga			BSES MG Hospital	India		

Supplemental Online Content: Nonauthor Collaborators

*Indicates required information. Only first name, last name, and suffix will appear in PubMed.

*First Name and Middle Initial(s)	*Last Name	*Suffix (eg, Jr, III)	Academic Degrees	Institution	Location (city, state/province, country)	Role or Contribution, eg, chair, principal investigator	Group (if more than 1 Group listed in the byline) and/or Subgroup (eg, Steering Committee)
Shilpa	Basety			BSES MG Hospital	India		
Shwetha	Dammareddy			BSES MG Hospital	India		
Phani Sreeharsha	Kasumalla			BSES MG Hospital	India		
Umamaheswara	Raju			Gandhi Medical College and Hospital,	India		
Janaki	Manduva			Gandhi Medical College and Hospital,	India		
Naresh	Kolakani			Gandhi Medical College and Hospital,	India		
Shreeja	Sripathi			Gandhi Medical College and Hospital,	India		
Sheetal	Chaitanya			Gandhi Medical College and Hospital,	India		
Anusha	Cherian			Jawaharlal Institute of Postgraduate I	India		
Sreejith	Parameswaran			Jawaharlal Institute of Postgraduate I	India		
Magesh	Parthiban			Jawaharlal Institute of Postgraduate I	India		
Priya A	Menu			Jawaharlal Institute of Postgraduate I	India		
Mardul Kumar	Daga			Maulana Azad Medical College and L	India		
Munisha	Agarwal			Maulana Azad Medical College and L	India		
Ishan	Rohtagi			Maulana Azad Medical College and L	India		
Sridhar	Papani			Medicover Hospitals	India		
Mahesh	Kamuram			Medicover Hospitals	India		
Kamlesh Kumar	Agrawal			Om Superspeciality Hospital	India		
Vijendra	Baghel			Om Superspeciality Hospital	India		
Kirti Kumar	Patel			Om Superspeciality Hospital	India		
Surapaneni Krishna	Mohan			Panimalar Medical College Hospital &	India		
Ekambaram	Jyothisree			Panimalar Medical College Hospital &	India		
Nooshin	Dalili			Labbafinejad Medical Center, Shahid	Iran		
Mohsen	Nafa			Labbafinejad Medical Center, Shahid	Iran		
Wataru	Matsuda			Center Hospital of the National Cente	Japan		
Reina	Suzuki			Jichi Medical University Saitama Med	Japan		
Masamitsu	Sanui			Jichi Medical University Saitama Med	Japan		
Sho	Horikita			Jichi Medical University Saitama Med	Japan		
Yuki	Itagaki			Sapporo City General Hospital	Japan		
Akira	Kodate			Sapporo City General Hospital	Japan		

Supplemental Online Content: Nonauthor Collaborators

*Indicates required information. Only first name, last name, and suffix will appear in PubMed.

*First Name and Middle Initial(s)	*Last Name	*Suffix (eg, Jr, III)	Academic Degrees	Institution	Location (city, state/province, country)	Role or Contribution, eg, chair, principal investigator	Group (if more than 1 Group listed in the byline) and/or Subgroup (eg, Steering Committee)
Yuki	Takahashi			Sapporo City General Hospital	Japan		
Koyo	Moriki			Sapporo City General Hospital	Japan		
Takuya	Shiga			Tohoku university hospital	Japan		
Yudai	Iwasaki			Tohoku university hospital	Japan		
Rene	Rodriguez-Gutierrez			Hospital Universitario, Universidad A	Mexico		
Jose Gerardo	Gonzale			Hospital Universitario, Universidad A	Mexico		
Alejandro	Salcido-Montenegro			Hospital Universitario, Universidad A	Mexico		
Adrian	Camacho-Ortiz			Hospital Universitario, Universidad A	Mexico		
Fatimah	Hassan-Hanga			Aminu Kano Teaching Hospital/Bayer	Nigeria		
Hadiza	Galadanci			Aminu Kano Teaching Hospital/Bayer	Nigeria		
Abubakar Shehu	Gezawa			Aminu Kano Teaching Hospital/Bayer	Nigeria		
Halima	Kabir			Aminu Kano Teaching Hospital/Bayer	Nigeria		
Taiwo Gboluwaga	Amole			Aminu Kano Teaching Hospital/Bayer	Nigeria		
Dalha Gwarzo	Haliru			Aminu Kano Teaching Hospital/Bayer	Nigeria		
Abdullahi S	Ibrahim			Aminu Kano Teaching Hospital/Bayer	Nigeria		
Muhammad Sohaib	Asghar			Dow University Hospital	Pakistan		
Mashaal	Syed			Dow University Hospital	Pakistan		
Syed Anosh Ali	Naqvi			Dow University Hospital	Pakistan		
Igor Borisovich	Zabolotskikh			Kuban State Medical University with	Russia		
Tatiana Sergeevna	Musaeva			Kuban State Medical University with	Russia		
Razan K	Alamoudi			King Fahad Armed Forces Hospital	Saudi Arabia		
Hassan M	AlSharif			King Fahad Armed Forces Hospital	Saudi Arabia		
Sarah A	Almazwaghi			King Fahad Armed Forces Hospital	Saudi Arabia		
Mohammed S	Elsakran			King Fahad Armed Forces Hospital	Saudi Arabia		
Mohamed A	Aid			King Fahad Armed Forces Hospital	Saudi Arabia		
Mouaz A	Darwich			King Fahad Armed Forces Hospital	Saudi Arabia		
Omnia M	Hagag			King Fahad Armed Forces Hospital	Saudi Arabia		
Salah A	Ali			King Fahad Armed Forces Hospital	Saudi Arabia		

*Indicates required information. Only first name, last name, and suffix will appear in PubMed.

*First Name and Middle Initial(s)	*Last Name	*Suffix (eg, Jr, III)	Academic Degrees	Institution	Location (city, state/province, country)	Role or Contribution, eg, chair, principal investigator	Group (if more than 1 Group listed in the byline) and/or Subgroup (eg, Steering Committee)
Alona	Rocacorba			King Fahad Armed Forces Hospital	Saudi Arabia		
Kathrine	Supena			King Fahad Armed Forces Hospital	Saudi Arabia		
Efren Ray	Juane			King Fahad Armed Forces Hospital	Saudi Arabia		
Jenalyn	Medina			King Fahad Armed Forces Hospital	Saudi Arabia		
Jowany	Baduria			King Fahad Armed Forces Hospital	Saudi Arabia		
Marwa Ridha	Amer			King Faisal Specialist Hospital & Resea	Saudi Arabia		
Mohammed Abdullah	Bawazeer			King Faisal Specialist Hospital & Resea	Saudi Arabia		
Talal I	Dahhan			King Faisal Specialist Hospital & Resea	Saudi Arabia		
Eiad	Kseibi			King Faisal Specialist Hospital & Resea	Saudi Arabia		
Abid Shahzad	Butt			King Faisal Specialist Hospital & Resea	Saudi Arabia		
Syed Moazzum	Khurshid			King Faisal Specialist Hospital & Resea	Saudi Arabia		
Muath	Rabee			King Faisal Specialist Hospital & Resea	Saudi Arabia		
Mohammed	Abujazar			King Faisal Specialist Hospital & Resea	Saudi Arabia		
Razan K	Alghunaim			King Faisal Specialist Hospital & Resea	Saudi Arabia		
Maal	Abualkhair			King Faisal Specialist Hospital & Resea	Saudi Arabia		
Abeer Turki	AlFirm			King Faisal Specialist Hospital & Resea	Saudi Arabia		
Mohammed A	Almazyad			King Saud University	Saudi Arabia		
Mohammed I	Alarifi			King Saud University	Saudi Arabia		
Jara M	Macarambon			King Saud University	Saudi Arabia		
Ahmad Abdullah	Bukhari			King Saud University	Saudi Arabia		
Hussian A	Albahrani			King Saud University	Saudi Arabia		
Kazi N	Asfina			King Saud University	Saudi Arabia		
Kaltham M	Aldossary			King Saud University	Saudi Arabia		
Marija Zdravkovic	Zoran			CHC Bezanijska kosa	Serbia		
Viseslaz	Popadic			CHC Bezanijska kosa	Serbia		
Slobodan	Klasnja			CHC Bezanijska kosa	Serbia		
Jovana	Bojicic			UMC Zvezdara	Serbia		
Bojan	Kovacevic			UMC Zvezdara	Serbia		
Stevanovic	Predrag			University Hospital Center "Dr Dragis	Serbia		
Dejan S	Stojakov			University Hospital Center "Dr Dragis	Serbia		

*Indicates required information. Only first name, last name, and suffix will appear in PubMed.

*First Name and Middle Initial(s)	*Last Name	*Suffix (eg, Jr, III)	Academic Degrees	Institution	Location (city, state/province, country)	Role or Contribution, eg, chair, principal investigator	Group (if more than 1 Group listed in the byline) and/or Subgroup (eg, Steering Committee)
Duska K	Ignjatovic			University Hospital Center "Dr Dragis	Serbia		
Suzana C	Bojicic			University Hospital Center "Dr Dragis	Serbia		
Mariana M	Bobos			University Hospital Center "Dr Dragis	Serbia		
Irina B	Nenadic			University Hospital Center "Dr Dragis	Serbia		
Milica S	Zaric			University Hospital Center "Dr Dragis	Serbia		
Marko D	Djuric			University Hospital Center "Dr Dragis	Serbia		
Vladimir R	Djukic			University Hospital Center "Dr Dragis	Serbia		
Santiago Y	Teruel			Hospital Universitario La Paz	Spain		
Belen C	Martin			Hospital Universitario La Paz	Spain		
Uluhan	Sili			Marmara Univeristy	Turkey		
Huseyin	Bilgin			Marmara Univeristy	Turkey		
Pinar	Ay			Marmara Univeristy	Turkey		
Kenneth W	Dodd			Advocate Christ Medical Center	United States of America		
Nicholas	Goodmanson			Advocate Christ Medical Center	United States of America		
Kathleen	Hesse			Advocate Christ Medical Center	United States of America		
Paige	Bird			Advocate Christ Medical Center	United States of America		
Chauncey	Weinert			Advocate Christ Medical Center	United States of America		
Nathan	Schoenrade			Advocate Christ Medical Center	United States of America		
Abdulrahman	Altaher			Advocate Christ Medical Center	United States of America		
Esmael	Mayar			Advocate Christ Medical Center	United States of America		
Matthew	Aronson			Advocate Christ Medical Center	United States of America		
Tyler	Cooper			Advocate Christ Medical Center	United States of America		
Monica	Logan			Advocate Christ Medical Center	United States of America		
Brianna	Miner			Advocate Christ Medical Center	United States of America		
Gisele	Papo			Advocate Christ Medical Center	United States of America		
Eric M	Siegal			Advocate/Aurora St Luke's Medical C	United States of America		
Phyllis	Runningen			Advocate/Aurora St Luke's Medical C	United States of America		
Love A	Patel			Allina Health (Abbott Northwestern H	United States of America		
Roman R	Melamed			Allina Health (Abbott Northwestern H	United States of America		
David M	Tierney			Allina Health (Abbott Northwestern H	United States of America		
Vino S	Raj			Allina Health (Abbott Northwestern H	United States of America		

*Indicates required information. Only first name, last name, and suffix will appear in PubMed.

*First Name and Middle Initial(s)	*Last Name	*Suffix (eg, Jr, III)	Academic Degrees	Institution	Location (city, state/province, country)	Role or Contribution, eg, chair, principal investigator	Group (if more than 1 Group listed in the byline) and/or Subgroup (eg, Steering Committee)
Narayana	Mazumder			Allina Health (Abbott Northwestern H	United States of America		
Catherine St	Hill			Allina Health (Abbott Northwestern H	United States of America		
Lisa	Kirkland			Allina Health (Abbott Northwestern H	United States of America		
Nova	Schmitz			Allina Health (Abbott Northwestern H	United States of America		
Alena	Sigman			Allina Health (Abbott Northwestern H	United States of America		
Joan	Hall			Allina Health (Abbott Northwestern H	United States of America		
Abhijit A	Raval			AnMed Health	United States of America		
Andrea	Franks			AnMed Health	United States of America		
Jennifer M	Jarvis			Ascension St. Mary's Hospital	United States of America		
Anmol	Kharbanda			Ascension St.Vincent Hospital	United States of America		
Sunil	Jhajhria			Ascension St.Vincent Hospital	United States of America		
Zachary	Fyffe			Ascension St.Vincent Hospital	United States of America		
Stephen	Capizzi			Ascension/St. Thomas Research Instit	United States of America		
Bethany	Alicie			Ascension/St. Thomas Research Instit	United States of America		
Martha	Green			Ascension/St. Thomas Research Instit	United States of America		
Lori	Corckarell			Ascension/St. Thomas Research Instit	United States of America		
Amelia	Drennan			Ascension/St. Thomas Research Instit	United States of America		
Kathleen	Dubuque			Ascension/St. Thomas Research Instit	United States of America		
Tonya	Fambrough			Ascension/St. Thomas Research Instit	United States of America		
Nikole	Gasaway			Ascension/St. Thomas Research Instit	United States of America		
Briana	Krantz			Ascension/St. Thomas Research Instit	United States of America		
Peiman	Nebi			Ascension/St. Thomas Research Instit	United States of America		
Jan	Orga			Ascension/St. Thomas Research Instit	United States of America		
Margaret	Serfass			Ascension/St. Thomas Research Instit	United States of America		
Alina	Simion			Ascension/St. Thomas Research Instit	United States of America		
Kimberly	Warren			Ascension/St. Thomas Research Instit	United States of America		
Cassie	Wheeler			Ascension/St. Thomas Research Instit	United States of America		
Cj	Woolman			Ascension/St. Thomas Research Instit	United States of America		
Amy B	Christie			Atrium Health Navicent	United States of America		
Dennis W	Ashley			Atrium Health Navicent	United States of America		
Rajani	Adiga			Atrium Health Navicent	United States of America		

*Indicates required information. Only first name, last name, and suffix will appear in PubMed.

*First Name and Middle Initial(s)	*Last Name	*Suffix (eg, Jr, III)	Academic Degrees	Institution	Location (city, state/province, country)	Role or Contribution, eg, chair, principal investigator	Group (if more than 1 Group listed in the byline) and/or Subgroup (eg, Steering Committee)
Andrew S	Moyer			Augusta Health	United States of America		
George M	Verghese			Augusta Health	United States of America		
Andrea	Sikora Newsome			Augusta University Medical Center	United States of America		
Chirsty C	Forehand			Augusta University Medical Center	United States of America		
Rebecca	Bruning			Augusta University Medical Center	United States of America		
Timothy W	Jones			Augusta University Medical Center	United States of America		
Moldovan	Sabov			Aultman Hospital	United States of America		
Fatema	Zaidi			Aultman Hospital	United States of America		
Fiona	Tissavirasingham			Aultman Hospital	United States of America		
Dhatri	Malipeddi			Aultman Hospital	United States of America		
Jarrod M	Mosier			Banner University Medical Center	United States of America		
Karen	Lutrick			Banner University Medical Center	United States of America		
Beth Salvagio	Campbell			Banner University Medical Center	United States of America		
Cathleen	Wilson			Banner University Medical Center	United States of America		
Patrick	Rivers			Banner University Medical Center	United States of America		
Jonathan	Brinks			Banner University Medical Center	United States of America		
Mokenge	Ndiva Mongoh			Banner University Medical Center	United States of America		
Boris	Gilson			Banner University Medical Center	United States of America		
Donaa Lee	Armaignac			Baptist Health South Florida	United States of America		
Don	Parris			Baptist Health South Florida	United States of America		
Maria Pilar	Zuniga			Baptist Health South Florida	United States of America		
Ilea	Vargas			Baptist Health South Florida	United States of America		
Viviana	Boronat			Baptist Health South Florida	United States of America		
Anneka	Hutton			Baptist Health South Florida	United States of America		
Navneet	Kaur			Baptist Health South Florida	United States of America		
Prashank	Neupane			Baptist Health South Florida	United States of America		
Nohemi	Sadule-Rios			Baptist Health South Florida	United States of America		
Lourdes M	Rojas			Baptist Health South Florida	United States of America		
Aashish	Neupane			Baptist Health South Florida	United States of America		

Supplemental Online Content: Nonauthor Collaborators

*Indicates required information. Only first name, last name, and suffix will appear in PubMed.

*First Name and Middle Initial(s)	*Last Name	*Suffix (eg, Jr, III)	Academic Degrees	Institution	Location (city, state/province, country)	Role or Contribution, eg, chair, principal investigator	Group (if more than 1 Group listed in the byline) and/or Subgroup (eg, Steering Committee)
Priscilla	Rivera			Baptist Health South Florida	United States of America		
Carlos	Valle Carlos			Baptist Health South Florida	United States of America		
Gregory	Vincent			Baptist Health South Florida	United States of America		
Mahesh	Amin			BayCare Health System	United States of America		
Mary E	Schelle			BayCare Health System	United States of America		
Amanda	Steadham			BayCare Health System	United States of America		
Christopher M	Howard			Baylor College of Medicine, Baylor St	United States of America		
Cameron	McBride			Baylor College of Medicine, Baylor St	United States of America		
Jocelyn	Abraham			Baylor College of Medicine, Baylor St	United States of America		
Orlando	Garner			Baylor College of Medicine, Baylor St	United States of America		
Katherine	Richards			Baylor College of Medicine, Baylor St	United States of America		
Keegan	Collins			Baylor College of Medicine, Baylor St	United States of America		
Preethi	Antony			Baylor College of Medicine, Baylor St	United States of America		
Sindhu	Mathew			Baylor College of Medicine, Baylor St	United States of America		
Valerie	Danesh			Baylor Scott & White Health	United States of America		
Gueorgui	Dubrocq			Baylor Scott & White Health	United States of America		
Amber L	Davis			Baylor Scott & White Health	United States of America		
Marissa J	Hammers			Baylor Scott & White Health	United States of America		
Ill M	McGahey			Baylor Scott & White Health	United States of America		
Amanda C	Farris			Baylor Scott & White Health	United States of America		
Elisa	Priest			Baylor Scott & White Health	United States of America		
Robyn	Korsmo			Baylor Scott & White Health	United States of America		
Lorie	Fares			Baylor Scott & White Health	United States of America		
Kathy	Skiles			Baylor Scott & White Health	United States of America		
Susan M	Shor			Baylor Scott & White Health	United States of America		
Kenya	Burns			Baylor Scott & White Health	United States of America		
Melody	Flores			Baylor Scott & White Health	United States of America		
Lindsay	Newman			Baylor Scott & White Health	United States of America		
Debora A	Wilk			Baylor Scott & White Health	United States of America		
Jason	Ettlinger			Baylor Scott & White Health	United States of America		
Jaccallene	Bomar			Baylor Scott & White Health	United States of America		

*Indicates required information. Only first name, last name, and suffix will appear in PubMed.

*First Name and Middle Initial(s)	*Last Name	*Suffix (eg, Jr, III)	Academic Degrees	Institution	Location (city, state/province, country)	Role or Contribution, eg, chair, principal investigator	Group (if more than 1 Group listed in the byline) and/or Subgroup (eg, Steering Committee)
Himani	Darji			Baylor Scott & White Health	United States of America		
Alejandro	Arroliga			Baylor Scott & White Health	United States of America		
Corrie A	Dowell			Baylor Scott & White Health	United States of America		
Gabriela Hope	Gonzales			Baylor Scott & White Health	United States of America		
Melody Flores	Flores			Baylor Scott & White Health	United States of America		
Lindsay	Newman			Baylor Scott & White Health	United States of America		
Allan j	Walkey			Boston University School of Medicine	United States of America		
Sushrut S	Waikar			Boston University School of Medicine	United States of America		
Michael A	Garcia			Boston University School of Medicine	United States of America		
Mia	Colona			Boston University School of Medicine	United States of America		
Zoe	Kibbelaar			Boston University School of Medicine	United States of America		
Michael	Leong			Boston University School of Medicine	United States of America		
Daniel	Wallman			Boston University School of Medicine	United States of America		
Kanupriya	Soni			Boston University School of Medicine	United States of America		
Jennifer	Maccarone			Boston University School of Medicine	United States of America		
Joshua	Gilman			Boston University School of Medicine	United States of America		
Ycar	Devis			Boston University School of Medicine	United States of America		
Joseph	Chung			Boston University School of Medicine	United States of America		
Munizay	Paracha			Boston University School of Medicine	United States of America		
David N	Lumelsky			Boston University School of Medicine	United States of America		
Madeline	DiLorenzo			Boston University School of Medicine	United States of America		
Najla	Abdurrahman			Boston University School of Medicine	United States of America		
Shelsey	Johnson			Boston University School of Medicine	United States of America		
Maj Andrew M	Hersh			Brooke Amry Medical Center	United States of America		
Stephanie L	Wachs			Brooke Amry Medical Center	United States of America		
Brittany S	Swigger			Brooke Amry Medical Center	United States of America		
Lauren A	Sattler			Brooke Amry Medical Center	United States of America		
Michael N	Moulton			Brooke Amry Medical Center	United States of America		
Kimberly	Zammit			Buffalo General Medical Center	United States of America		
Patrick J	McGrath			Buffalo General Medical Center	United States of America		
William	Loeffler			Buffalo General Medical Center	United States of America		

Supplemental Online Content: Nonauthor Collaborators

*Indicates required information. Only first name, last name, and suffix will appear in PubMed.

*First Name and Middle Initial(s)	*Last Name	*Suffix (eg, Jr, III)	Academic Degrees	Institution	Location (city, state/province, country)	Role or Contribution, eg, chair, principal investigator	Group (if more than 1 Group listed in the byline) and/or Subgroup (eg, Steering Committee)
Maya R	Chilbert			Buffalo General Medical Center	United States of America		
Raghavendra	Tirupathi			Chambersburg Hospital	United States of America		
Alymer	Tang			Chambersburg Hospital	United States of America		
Arshad	Safi			Chambersburg Hospital	United States of America		
Cindy	Green			Chambersburg Hospital	United States of America		
Jackie	Newell			Chambersburg Hospital	United States of America		
Naga	Ramani			Chambersburg Hospital	United States of America		
Bhavani Harika	Ganti			Chambersburg Hospital	United States of America		
Rayan E	Ihle			Charleston Area Medical Center	United States of America		
Elaine A	Davis			Charleston Area Medical Center	United States of America		
Shelda A	Martin			Charleston Area Medical Center	United States of America		
Imran A	Sayed			Children's Hosptial Colorado, Univers	United States of America		
Katja M	Gist			Children's Hosptial Colorado, Univers	United States of America		
Larisa	Strom			Children's Hosptial Colorado, Univers	United States of America		
Kathleen	Chiotos			Children's Hosptial Colorado, Univers	United States of America		
Allison M	Blatz			Children's Hospital of Philadelphia	United States of America		
Giyoung	Lee			Children's Hospital of Philadelphia	United States of America		
Ryan H	Burnett			Children's Hospital of Philadelphia	United States of America		
Guy I	Sydney			Children's Hospital of Philadelphia	United States of America		
Danielle M	Traynor			Children's Hospital of Philadelphia	United States of America		
Salim	Surani			Christus Spohn Shoreline Corpus Chri	United States of America		
Joshua	White			Christus Spohn Shoreline Corpus Chri	United States of America		
Aftab	Khan			Christus Spohn Shoreline Corpus Chri	United States of America		
Rahul	Dhahwal			Christus Spohn Shoreline Corpus Chri	United States of America		
Sreekanth	Cheruku			Clement Universit Hospital at UT Sou	United States of America		
Farzin	Ahmed			Clement Universit Hospital at UT Sou	United States of America		
Chrispoter	Deonarine			Clement Universit Hospital at UT Sou	United States of America		
Ashley	Jones			Clement Universit Hospital at UT Sou	United States of America		
Mohammad Ali	Shaikh			Clement Universit Hospital at UT Sou	United States of America		
David	Preston			Clement Universit Hospital at UT Sou	United States of America		
Jeanette	Chin			Clement Universit Hospital at UT Sou	United States of America		

Supplemental Online Content: Nonauthor Collaborators

*Indicates required information. Only first name, last name, and suffix will appear in PubMed.

***First Name and Middle Initial(s)**	***Last Name**	***Suffix (eg, Jr, III)**	Academic Degrees	Institution	Location (city, state/province, country)	Role or Contribution, eg, chair, principal investigator	Group (if more than 1 Group listed in the byline) and/or Subgroup (eg, Steering Committee)
Vidula	Vachharajani			Cleveland Clinic (Main Campus, Fairv	United States of America		
Abhijit	Duggal			Cleveland Clinic (Main Campus, Fairv	United States of America		
Prabalini	Rajendram			Cleveland Clinic (Main Campus, Fairv	United States of America		
Omar	Mehkri			Cleveland Clinic (Main Campus, Fairv	United States of America		
Siddharth	Dugar			Cleveland Clinic (Main Campus, Fairv	United States of America		
Michelle	Biehl			Cleveland Clinic (Main Campus, Fairv	United States of America		
Gretchen	Sacha			Cleveland Clinic (Main Campus, Fairv	United States of America		
Stuart	Houltham			Cleveland Clinic (Main Campus, Fairv	United States of America		
Alexander	Kind			Cleveland Clinic (Main Campus, Fairv	United States of America		
Kiran	Ashok			Cleveland Clinic (Main Campus, Fairv	United States of America		
Bryan	Poynter			Cleveland Clinic (Main Campus, Fairv	United States of America		
Mary E	Beukemann			Cleveland Clinic (Main Campus, Fairv	United States of America		
Richard	Rice			Cleveland Clinic (Main Campus, Fairv	United States of America		
Susan	Gole			Cleveland Clinic (Main Campus, Fairv	United States of America		
Valerie	Shaner			Cleveland Clinic (Main Campus, Fairv	United States of America		
Adarsh	Conjeevaram			Cleveland Clinic (Main Campus, Fairv	United States of America		
Michlelle	Ferrari			Cleveland Clinic (Main Campus, Fairv	United States of America		
Narendrakumar	Alappan			Cleveland Clinic (Main Campus, Fairv	United States of America		
Steven	Minear			Cleveland Clinic (Main Campus, Fairv	United States of America		
Jaime	Hernandez-Montfort			Cleveland Clinic (Main Campus, Fairv	United States of America		
Syed Sohaib	Nasim			Cleveland Clinic (Main Campus, Fairv	United States of America		
Ravi	Sunderkrishnan			Cleveland Clinic (Main Campus, Fairv	United States of America		
Debasis	Sahoo			Cleveland Clinic (Main Campus, Fairv	United States of America		
Patrick S	Milligan			Community Health Network Indianap	United States of America		
Sandeep K	Gupta			Community Health Network Indianap	United States of America		
Joy M	Koglin			Community Health Network Indianap	United States of America		
Regina	Gibson			Community Health Network Indianap	United States of America		
Lana	Johnson			Community Health Network Indianap	United States of America		
Felicia	Preston			Community Health Network Indianap	United States of America		

Supplemental Online Content: Nonauthor Collaborators

*Indicates required information. Only first name, last name, and suffix will appear in PubMed.

*First Name and Middle Initial(s)	*Last Name	*Suffix (eg, Jr, III)	Academic Degrees	Institution	Location (city, state/province, country)	Role or Contribution, eg, chair, principal investigator	Group (if more than 1 Group listed in the byline) and/or Subgroup (eg, Steering Committee)
Crimson	Scott			Community Health Network Indianap	United States of America		
Bethany	Nungester			Community Health Network Indianap	United States of America		
Dana D	Byrne			Cooper Univeristy Health Care	United States of America		
Christa A	Schorr			Cooper Univeristy Health Care	United States of America		
Katie	Grant			Cooper Univeristy Health Care	United States of America		
Katherine L	Doktar			Cooper Univeristy Health Care	United States of America		
Maura C	Porto			Cooper Univeristy Health Care	United States of America		
Olgo	Kaplan			Cooper Univeristy Health Care	United States of America		
James E	Siegler			Cooper Univeristy Health Care	United States of America		
Brian	Schonewald			Cooper Univeristy Health Care	United States of America		
Ashley	Woodford			Cooper Univeristy Health Care	United States of America		
Alan	Tsai			Cooper Univeristy Health Care	United States of America		
Savina	Reid			Cooper Univeristy Health Care	United States of America		
Kuntal	Bhowmick			Cooper Univeristy Health Care	United States of America		
Saba	Daneshpooy			Cooper Univeristy Health Care	United States of America		
Cyrus	Mowdawalla			Cooper Univeristy Health Care	United States of America		
Trishna Akshay	Dave			Cooper Univeristy Health Care	United States of America		
Wilhemina Kennedy	Connor Crudeli			Cooper Univeristy Health Care	United States of America		
Chirstopher	Ferry			Cooper Univeristy Health Care	United States of America		
Long	Nguyen			Cooper Univeristy Health Care	United States of America		
Sneha	Modi			Cooper Univeristy Health Care	United States of America		
Niharika	Padala			Cooper Univeristy Health Care	United States of America		
Pavan Jitendra	Patel			Cooper Univeristy Health Care	United States of America		
Belle	Lin			Cooper Univeristy Health Care	United States of America		
Jamie	Qiuyun			Cooper Univeristy Health Care	United States of America		
Fan Mandi	Liu			Cooper Univeristy Health Care	United States of America		
Rasagna	Kota			Cooper Univeristy Health Care	United States of America		
Annesha	Banerjee			Cooper Univeristy Health Care	United States of America		
Steven K	Daugherty			Coz Medical Center Springfield	United States of America		
Sam	Atkinson			Coz Medical Center Springfield	United States of America		

*Indicates required information. Only first name, last name, and suffix will appear in PubMed.

*First Name and Middle Initial(s)	*Last Name	*Suffix (eg, Jr, III)	Academic Degrees	Institution	Location (city, state/province, country)	Role or Contribution, eg, chair, principal investigator	Group (if more than 1 Group listed in the byline) and/or Subgroup (eg, Steering Committee)
Kelly	Shrimpton			Coz Medical Center Springfield	United States of America		
Sidney	Ontai			Detar Family Medicine Residency	United States of America		
Brian	Contreras			Detar Family Medicine Residency	United States of America		
Uzoma	Obinwanko			Detar Family Medicine Residency	United States of America		
Nneka	Amamasi			Detar Family Medicine Residency	United States of America		
Amir	Sharafi			Detar Family Medicine Residency	United States of America		
Sarah	Lee			Detroit Medical Centre	United States of America		
Zahia	Esber			Detroit Medical Centre	United States of America		
Chetna	Jinjvadia			Detroit Medical Centre	United States of America		
Raquel R	Bartz			Duke University Hospital	United States of America		
Vijay	Krishnamoorthy			Duke University Hospital	United States of America		
Bryan	Kraft			Duke University Hospital	United States of America		
Aaron	Pulsipher			Duke University Hospital	United States of America		
Eugene	Friedman			Duke University Hospital	United States of America		
Sachin	Mehta			Duke University Hospital	United States of America		
Margit	Kaufman			Englewood Health	United States of America		
Gregg	Lobel			Englewood Health	United States of America		
Nisha	Gandhi			Englewood Health	United States of America		
Amr	Abdelaty			Englewood Health	United States of America		
Elizabeth	Shaji			Englewood Health	United States of America		
Kiana	Lim			Englewood Health	United States of America		
Juan	Marte			Englewood Health	United States of America		
Dani Ashley	Sosa			Englewood Health	United States of America		
David P	Yamane			Geroge Washington University	United States of America		
Ivy	Benjenk			Geroge Washington University	United States of America		
Nivedita	Prasanna			Geroge Washington University	United States of America		
Nicholas	Perkins			Greenville Memorial Hospital	United States of America		
Prera J	Roth			Greenville Memorial Hospital	United States of America		
Alain	Litwin			Greenville Memorial Hospital	United States of America		
Ananda	Pariyadath			Greenville Memorial Hospital	United States of America		

Supplemental Online Content: Nonauthor Collaborators

*Indicates required information. Only first name, last name, and suffix will appear in PubMed.

*First Name and Middle Initial(s)	*Last Name	*Suffix (eg, Jr, III)	Academic Degrees	Institution	Location (city, state/province, country)	Role or Contribution, eg, chair, principal investigator	Group (if more than 1 Group listed in the byline) and/or Subgroup (eg, Steering Committee)
Phillip	Moschella			Greenville Memorial Hospital	United States of America		
Trayson	Llano			Greenville Memorial Hospital	United States of America		
Christine	Waller			Gundersen Health System La Crosse \	United States of America		
Kara	Kallies			Gundersen Health System La Crosse \	United States of America		
Christine	Waller			Gundersen Health System La Crosse \	United States of America		
Jonean	Thorsen			Gundersen Health System La Crosse \	United States of America		
Alec	Fitzsimmons			Gundersen Health System La Crosse \	United States of America		
Haley	Olsen			Gundersen Health System La Crosse \	United States of America		
Normal	Smalls			Howard University Hospital	United States of America		
Steven Q	Davis			JPS Health Network	United States of America		
Valentina	Jovic			JPS Health Network	United States of America		
Max	Masuda			JPS Health Network	United States of America		
Amanda	Hayes			JPS Health Network	United States of America		
Katharine	Nault			Lahey Hospital & Medical Center	United States of America		
Michael	Smith			Lakes Region General Hospital	United States of America		
William	Snow			Lakes Region General Hospital	United States of America		
Riley	Liptak			Lakes Region General Hospital	United States of America		
Hannah	Durant			Lakes Region General Hospital	United States of America		
Valerie	Pendleton			Lakes Region General Hospital	United States of America		
Alay	Nanavati			Lakes Region General Hospital	United States of America		
Risa	Mrozowsk			Lakes Region General Hospital	United States of America		
Erica	Doubleday			Lakes Region General Hospital	United States of America		
Yuk Min	Liu			Loyola University Medical Center	United States of America		
Sarah	Zavala			Loyola University Medical Center	United States of America		
Esther	Shim			Loyola University Medical Center	United States of America		
Ronald A	Reilkoff			M Health - Fairview	United States of America		
Julia A	Heneghan			M Health - Fairview	United States of America		
Sarah	Eichen			M Health - Fairview	United States of America		
Lexie	Goertzen			M Health - Fairview	United States of America		
Scott	Rajala			M Health - Fairview	United States of America		
Ghislaine	Feussom			M Health - Fairview	United States of America		

Supplemental Online Content: Nonauthor Collaborators

*Indicates required information. Only first name, last name, and suffix will appear in PubMed.

***First Name and Middle Initial(s)**	***Last Name**	***Suffix (eg, Jr, III)**	Academic Degrees	Institution	Location (city, state/province, country)	Role or Contribution, eg, chair, principal investigator	Group (if more than 1 Group listed in the byline) and/or Subgroup (eg, Steering Committee)
Ben	Tang			M Health - Fairview	United States of America		
Christine C	Junia			MacNeal Hospital Loyola Medicine	United States of America		
Robert	Lichtenberg			MacNeal Hospital Loyola Medicine	United States of America		
Hasrat	Sidhu			MacNeal Hospital Loyola Medicine	United States of America		
Diana	Espinoza			MacNeal Hospital Loyola Medicine	United States of America		
Shelden	Rodrigues			MacNeal Hospital Loyola Medicine	United States of America		
Marai Jose	Zabala			MacNeal Hospital Loyola Medicine	United States of America		
Daniela	Goyes			MacNeal Hospital Loyola Medicine	United States of America		
Ammu	Susheela			MacNeal Hospital Loyola Medicine	United States of America		
Buddhi	Hatharaliyadda			MacNeal Hospital Loyola Medicine	United States of America		
Naveen	Rameshkumar			MacNeal Hospital Loyola Medicine	United States of America		
Amulya	Kasireddy			MacNeal Hospital Loyola Medicine	United States of America		
Genessis	Maldonado			MacNeal Hospital Loyola Medicine	United States of America		
Lisseth	Beltran			MacNeal Hospital Loyola Medicine	United States of America		
Akshata	Chaugule			MacNeal Hospital Loyola Medicine	United States of America		
Hassan	Khan			MacNeal Hospital Loyola Medicine	United States of America		
Namrata	Patil			Mass General Brigham Hospital	United States of America		
Ruhi	Patil			Mass General Brigham Hospital	United States of America		
Rodrigo	Cartin-Ceba			Mayo Clinic Arizona	United States of America		
Ayan	Sen			Mayo Clinic Arizona	United States of America		
Fahimeh	Talaei			Mayo Clinic Arizona	United States of America		
Rahul	Kashyap			Mayo Clinic Rochester	United States of America		
Juan	Pablo Domecq			Mayo Clinic Rochester	United States of America		
Ognjen	Gajic			Mayo Clinic Rochester	United States of America		
Vikas	Bansal			Mayo Clinic Rochester	United States of America		
Aysun	Tekin			Mayo Clinic Rochester	United States of America		
Amos	Lal			Mayo Clinic Rochester	United States of America		
John C	O'Horo			Mayo Clinic Rochester	United States of America		
Neha N	Deo			Mayo Clinic Rochester	United States of America		
Mayank	Sharma			Mayo Clinic Rochester	United States of America		
Shahraz	Qamar			Mayo Clinic Rochester	United States of America		

*Indicates required information. Only first name, last name, and suffix will appear in PubMed.

***First Name and Middle Initial(s)**	***Last Name**	***Suffix (eg, Jr, III)**	Academic Degrees	Institution	Location (city, state/province, country)	Role or Contribution, eg, chair, principal investigator	Group (if more than 1 Group listed in the byline) and/or Subgroup (eg, Steering Committee)
Romil	Singh			Mayo Clinic Rochester	United States of America		
Diana J	Valencia Morales			Mayo Clinic Rochester	United States of America		
Abigail T	La Nou			Mayo Clinic Eau Claire	United States of America		
Marija	Bogojevic			Mayo Clinic Eau Claire	United States of America		
Simon	Zec			Mayo Clinic Eau Claire	United States of America		
Devang	Sanghavi			Mayo Clinic Florida	United States of America		
Pramod	Guru			Mayo Clinic Florida	United States of America		
Pablo	Morno Franco			Mayo Clinic Florida	United States of America		
Karthik	Ganaphadithan			Mayo Clinic Florida	United States of America		
Hollie	Saunders			Mayo Clinic Florida	United States of America		
Zachary	Fleissner			Mayo Clinic Florida	United States of America		
Juan	Garcia			Mayo Clinic Florida	United States of America		
Alejandra	Yu Lee Mateus			Mayo Clinic Florida	United States of America		
Siva Naga	Yarrarapu			Mayo Clinic Florida	United States of America		
Nirmaljot	Kaur			Mayo Clinic Florida	United States of America		
Abhisekh	Giri			Mayo Clinic Florida	United States of America		
Mohammed	Mustafa Hasan			Mayo Clinic Florida	United States of America		
Ashrita	Donepudi			Mayo Clinic Florida	United States of America		
Syed Anjum	Khan			Mayo clinic Mankato	United States of America		
Nitesh Kumar	Jain			Mayo clinic Mankato	United States of America		
Thoyaja	Koritala			Mayo clinic Mankato	United States of America		
Rahul S	Nanchal			Medical college of Wisconsin	United States of America		
Paul A	Bergl			Medical college of Wisconsin	United States of America		
Jennifer L	Peterson			Medical college of Wisconsin	United States of America		
Travis	Yamanaka			Mercy Hospital and Medical Center	United States of America		
Nicholas A	Barreras			Mercy Hospital and Medical Center	United States of America		
Michael	Markos			Mercy Hospital and Medical Center	United States of America		
Anita	Fareeduddin			Mercy Hospital and Medical Center	United States of America		
Rohan	Mehta			Mercy Hospital and Medical Center	United States of America		

Supplemental Online Content: Nonauthor Collaborators

*Indicates required information. Only first name, last name, and suffix will appear in PubMed.

*First Name and Middle Initial(s)	*Last Name	*Suffix (eg, Jr, III)	Academic Degrees	Institution	Location (city, state/province, country)	Role or Contribution, eg, chair, principal investigator	Group (if more than 1 Group listed in the byline) and/or Subgroup (eg, Steering Committee)
Chakradhar	Venkata			Mercy Hospital, Saint Louis	United States of America		
Miriam	Engemann			Mercy Hospital, Saint Louis	United States of America		
Annamarie	Mantese			Mercy Hospital, Saint Louis	United States of America		
Yasir	Tarabichi			MetroHealth Medical Center	United States of America		
Adam	Perzynski			MetroHealth Medical Center	United States of America		
Christine	Wang			MetroHealth Medical Center	United States of America		
Dhatri	Kotekal			MetroHealth Medical Center	United States of America		
Adriana C	Briceno Bierwirth			Midland Memorial Hospital, Texas Te	United States of America		
Gabriela M	Orellana			Midland Memorial Hospital, Texas Te	United States of America		
Gerardo	Catalasan			Midland Memorial Hospital, Texas Te	United States of America		
Shohana	Ahmed			Midland Memorial Hospital, Texas Te	United States of America		
Carlos F	Matute			Midland Memorial Hospital, Texas Te	United States of America		
Ahmad	Hamdan			Midland Memorial Hospital, Texas Te	United States of America		
Ivania	Salinas			Midland Memorial Hospital, Texas Te	United States of America		
Genesis Del	Nogal			Midland Memorial Hospital, Texas Te	United States of America		
Angel	Tejada			Midland Memorial Hospital, Texas Te	United States of America		
Jen-Ting	Chen			Montefiore Medical Center	United States of America		
Aluko	Hope			Montefiore Medical Center	United States of America		
Zoe	Tsagaris			Montefiore Medical Center	United States of America		
Elise	Ruen			Montefiore Medical Center	United States of America		
Aram	Hambardzumya n			Montefiore Medical Center	United States of America		
Nasar A	Siddiqi			New Hanover Regional Medical Cente	United States of America		
Lesly	Jurado			New Hanover Regional Medical Cente	United States of America		
Lindsey	Tincher			New Hanover Regional Medical Cente	United States of America		
Carolyn	Brown			New Hanover Regional Medical Cente	United States of America		
Bhagat S	Aulakh			OSF Saint Francis Medical Center	United States of America		
Sandeep	Tripathi			OSF Saint Francis Medical Center	United States of America		
Jennifer A	Bandy			OSF Saint Francis Medical Center	United States of America		
Lisa	Kreps			OSF Saint Francis Medical Center	United States of America		

*Indicates required information. Only first name, last name, and suffix will appear in PubMed.

*First Name and Middle Initial(s)	*Last Name	*Suffix (eg, Jr, III)	Academic Degrees	Institution	Location (city, state/province, country)	Role or Contribution, eg, chair, principal investigator	Group (if more than 1 Group listed in the byline) and/or Subgroup (eg, Steering Committee)
Dwan R	Bollinger			OSF Saint Francis Medical Center	United States of America		
Roger	Scott Stienecker			Parkview Health System	United States of America		
Andre G	Melendez			Parkview Health System	United States of America		
Tressa A	Brunner			Parkview Health System	United States of America		
Sue M	Budzon			Parkview Health System	United States of America		
Jessica L	Heffernan			Parkview Health System	United States of America		
Janelle M	Souder			Parkview Health System	United States of America		
Tracy L	Miller			Parkview Health System	United States of America		
Andrea G	Maisonneuve			Parkview Health System	United States of America		
Roberta E	Redfern			ProMedica Toleda Hospital	United States of America		
Jessica	Shoemaker			ProMedica Toleda Hospital	United States of America		
Jennifer	Micham			ProMedica Toleda Hospital	United States of America		
Lynn	Kenney			ProMedica Toleda Hospital	United States of America		
Gabriela	Naimy			ProMedica Toleda Hospital	United States of America		
Kerry P	J Pulver			Saint Alphonsus Regional Medical Cer	United States of America		
Jennifer	Yehle			Saint Alphonsus Regional Medical Cer	United States of America		
Alicia	Weeks			Saint Alphonsus Regional Medical Cer	United States of America		
Terra	Inman			Saint Alphonsus Regional Medical Cer	United States of America		
Brain L	Delmonaco			Samaritan Health Services	United States of America		
Anthony	Franklin			Samaritan Health Services	United States of America		
Mitchell	Heath			Samaritan Health Services	United States of America		
Antonia L	Vilella			Sarasota Memorial Hospital	United States of America		
Sara B	Kutner			Sarasota Memorial Hospital	United States of America		
Kacie	Clark			Sarasota Memorial Hospital	United States of America		
Danielle	Moore			Sarasota Memorial Hospital	United States of America		
Harry L	Anderson			St. Joseph Mercy Ann Arbor	United States of America		
Dixy	Rajkumar			St. Joseph Mercy Ann Arbor	United States of America		
Ali	Abunayla			St. Joseph Mercy Ann Arbor	United States of America		
Jerrilyn	Heiter			St. Joseph Mercy Ann Arbor	United States of America		
Howard A	Zaren			St. Joseph's Candler Health System	United States of America		

Supplemental Online Content: Nonauthor Collaborators

*Indicates required information. Only first name, last name, and suffix will appear in PubMed.

***First Name and Middle Initial(s)**	***Last Name**	***Suffix (eg, Jr, III)**	Academic Degrees	Institution	Location (city, state/province, country)	Role or Contribution, eg, chair, principal investigator	Group (if more than 1 Group listed in the byline) and/or Subgroup (eg, Steering Committee)
Stephanie J	Smith			St. Joseph's Candler Health System	United States of America		
Grant C	Lewis			St. Joseph's Candler Health System	United States of America		
Lauren	Seames			St. Joseph's Candler Health System	United States of America		
Cheryl	Farlow			St. Joseph's Candler Health System	United States of America		
Judy	Miller			St. Joseph's Candler Health System	United States of America		
Gloria	Broadstreet			St. Joseph's Candler Health System	United States of America		
Anthony	Martinez			St. Agnes Hospital	United States of America		
Micheal	Allison			St. Agnes Hospital	United States of America		
Aniket	Mittal			St. Agnes Hospital	United States of America		
Rafael	Ruiz			St. Agnes Hospital	United States of America		
Aleta	Skaanland			St. Agnes Hospital	United States of America		
Robert	Ross			St. Agnes Hospital	United States of America		
Umang	Patel			St. Mary Medical Center	United States of America		
Jordesha	Hodge			St. Mary Medical Center	United States of America		
Krunal Kumar	Patel			St. Mary Medical Center	United States of America		
Shivani	Dalal			St. Mary Medical Center	United States of America		
Himanshu	Kavani			St. Mary Medical Center	United States of America		
Sam	Joseph			St. Mary Medical Center	United States of America		
Michael A	Bernstein			Stamdford Health	United States of America		
Ian K	Goff			Stamdford Health	United States of America		
Matthew	Naftilan			Stamdford Health	United States of America		
Amal	Mathew			Stamdford Health	United States of America		
Debora	Williams			Stamdford Health	United States of America		
Sue	Murdock			Stamdford Health	United States of America		
Maryanne	Ducey			Stamdford Health	United States of America		
Kerianne	Nelson			Stamdford Health	United States of America		
Jason	Block			Standford Hospital and Clinics	United States of America		
James	Mitchel			Standford Hospital and Clinics	United States of America		
Connor G	O'Brien			Standford Hospital and Clinics	United States of America		
Sylvan	Cox			Standford Hospital and Clinics	United States of America		
Ioana	Amzuta			SUNY Update Medical Univeristy	United States of America		

Supplemental Online Content: Nonauthor Collaborators

*Indicates required information. Only first name, last name, and suffix will appear in PubMed.

***First Name and Middle Initial(s)**	***Last Name**	***Suffix (eg, Jr, III)**	Academic Degrees	Institution	Location (city, state/province, country)	Role or Contribution, eg, chair, principal investigator	Group (if more than 1 Group listed in the byline) and/or Subgroup (eg, Steering Committee)
Amish	Shah			SUNY Update Medical Univeristy	United States of America		
Ritu	Modi			SUNY Update Medical Univeristy	United States of America		
Hassan	Al-Khalisy			SUNY Update Medical Univeristy	United States of America		
Pradeep	Masuta			SUNY Update Medical Univeristy	United States of America		
Melissa	Schafer			SUNY Update Medical Univeristy	United States of America		
Angela	Wratney			SUNY Update Medical Univeristy	United States of America		
Kristina L	Carter			Texas Health Resources	United States of America		
Michael	Olmos			Texas Health Resources	United States of America		
Brittany M	Parker			Texas Health Resources	United States of America		
Julio	Quintanilla			Texas Health Resources	United States of America		
Tara A	Craig			Texas Health Resources	United States of America		
Brendon J	Clough			Texas Health Resources	United States of America		
Jeffrey T	Jameson			Texas Health Resources	United States of America		
Neha	Gupta			The Children's Hospital at OU Medicii	United States of America		
Tacy L	Jones			The Children's Hospital at OU Medicii	United States of America		
Shonda C	Ayers			The Children's Hospital at OU Medicii	United States of America		
Amy B	Harrell			The Children's Hospital at OU Medicii	United States of America		
Brent R	Brown			The Children's Hospital at OU Medicii	United States of America		
Caleb	Darby			The University of Tennessee Medical	United States of America		
Kristy	Page			The University of Tennessee Medical	United States of America		
Amanda	Brown			The University of Tennessee Medical	United States of America		
Jessie	McAbee			The University of Tennessee Medical	United States of America		
Katherine A	Belden			Thomas Jefferson University Hospital	United States of America		
Michael	Baram			Thomas Jefferson University Hospital	United States of America		
Devin M	Weber			Thomas Jefferson University Hospital	United States of America		
Rosalie	DePaola			Thomas Jefferson University Hospital	United States of America		
Yuwei	Xia			Thomas Jefferson University Hospital	United States of America		
Hudson	Carter			Thomas Jefferson University Hospital	United States of America		
Aaron	Tolley			Thomas Jefferson University Hospital	United States of America		
Mary	Ferranti			Thomas Jefferson University Hospital	United States of America		
Mark	Steele			Truman Medical Centers	United States of America		

*Indicates required information. Only first name, last name, and suffix will appear in PubMed.

*First Name and Middle Initial(s)	*Last Name	*Suffix (eg, Jr, III)	Academic Degrees	Institution	Location (city, state/province, country)	Role or Contribution, eg, chair, principal investigator	Group (if more than 1 Group listed in the byline) and/or Subgroup (eg, Steering Committee)
Laurie	Kemble			Truman Medical Centers	United States of America		
Joshua	Sethi			Tulane University Medical Center and	United States of America		
Chung	Cheng Han			Tulane University Medical Center and	United States of America		
Jaclyn	Pagliaro			Tulane University Medical Center and	United States of America		
Abdurrahman	Husian			UC San Diego Medical Center	United States of America		
Atul	Malhotra			UC San Diego Medical Center	United States of America		
Qais	Zawaydeh			UC San Diego Medical Center	United States of America		
Benjamin J	Sines			UNC Medical Center	United States of America		
Thomas J	Bice			UNC Medical Center	United States of America		
Dragana	Markotic			University Clinical Hospital, Mostar, B	United States of America		
Ivana	Bosnjak			University Clinical Hospital, Mostar, B	United States of America		
Emily A	Vail			University Hospital San Antonio	United States of America		
Susannah	Nicholson			University Hospital San Antonio	United States of America		
Rachelle B	Jonas			University Hospital San Antonio	United States of America		
AnnaRose E	Dement			University Hospital San Antonio	United States of America		
William	Tang			University Hospital San Antonio	United States of America		
Mark	DeRose			University Hospital San Antonio	United States of America		
Robert E	Villarreal			University Hospital San Antonio	United States of America		
Rajany V	Dy			University Medical Center of Souther	United States of America		
Alfredo	Lardino			University Medical Center of Souther	United States of America		
Jill	Sharma			University Medical Center of Souther	United States of America		
Richard	Czieki			University Medical Center of Souther	United States of America		
Julia	Christopher			University Medical Center of Souther	United States of America		
Ryan	Lacey			University Medical Center of Souther	United States of America		
Marwan	Mashina			University Medical Center of Souther	United States of America		
Kushal	Patel			University Medical Center of Souther	United States of America		
Dina	Gomaa			University of Cincinnati	United States of America		
Micahel	Goodman			University of Cincinnati	United States of America		
Devin	Wakefield			University of Cincinnati	United States of America		
Anthony	Spuzzillo			University of Cincinnati	United States of America		
John O	Shinn II			University of Cincinnati	United States of America		

Supplemental Online Content: Nonauthor Collaborators

*Indicates required information. Only first name, last name, and suffix will appear in PubMed.

***First Name and Middle Initial(s)**	***Last Name**	***Suffix (eg, Jr, III)**	Academic Degrees	Institution	Location (city, state/province, country)	Role or Contribution, eg, chair, principal investigator	Group (if more than 1 Group listed in the byline) and/or Subgroup (eg, Steering Committee)
Azra	Bihorac			University of Florida Health Shands H	United States of America		
Tezcan	Ozrazgat Baslanti			University of Florida Health Shands H	United States of America		
George	Omalay			University of Florida Health Shands H	United States of America		
Haleh	Hashemighouchani			University of Florida Health Shands H	United States of America		
Julie S	Cupka			University of Florida Health Shands H	United States of America		
Matthew M	Ruppert			University of Florida Health Shands H	United States of America		
Patrick W	McGonagill			University of Iowa Carver College of M	United States of America		
Colette	Galet			University of Iowa Carver College of M	United States of America		
Janice	Hubbard			University of Iowa Carver College of M	United States of America		
David	Wang			University of Iowa Carver College of M	United States of America		
Lauren	Allan			University of Iowa Carver College of M	United States of America		
Aditya	Badheka			University of Iowa Carver College of M	United States of America		
Madhuradhar	Chegondi			University of Iowa Carver College of M	United States of America		
Usman	Nazir			University of Kansas Medical Center	United States of America		
Garrett	Rampon			University of Kansas Medical Center	United States of America		
Jake	Riggle			University of Kansas Medical Center	United States of America		
Nathan	Dismag			University of Kansas Medical Center	United States of America		
Ozan	Akca			University of Louisville Hospital	United States of America		
Rainer	Lenhardt			University of Louisville Hospital	United States of America		
Rodrigo S	Cavallazzi			University of Louisville Hospital	United States of America		
Ann	Jerde			University of Louisville Hospital	United States of America		
Alexa	Black			University of Louisville Hospital	United States of America		
Allison	Polidori			University of Louisville Hospital	United States of America		
Haily	Griffey			University of Louisville Hospital	United States of America		
Justin	Winkler			University of Louisville Hospital	United States of America		
Thomas	Brenzel			University of Louisville Hospital	United States of America		
Rodger A	Alvarez			Univeristy of Maiami Miller School of	United States of America		
Amarilys	Alarocon-Calderon			Univeristy of Maiami Miller School of	United States of America		

*Indicates required information. Only first name, last name, and suffix will appear in PubMed.

***First Name and Middle Initial(s)**	***Last Name**	***Suffix (eg, Jr, III)**	Academic Degrees	Institution	Location (city, state/province, country)	Role or Contribution, eg, chair, principal investigator	Group (if more than 1 Group listed in the byline) and/or Subgroup (eg, Steering Committee)
Marie Anne	Sosa			Univeristy of Maiami Miller School of	United States of America		
Sunita K	Mahabir			Univeristy of Maiami Miller School of	United States of America		
Mausam J	Patel			Univeristy of Maiami Miller School of	United States of America		
Pauline	Parker			Univeristy of Michigan Health System	United States of America		
Andre	Admon			Univeristy of Michigan Health System	United States of America		
Sinan	Hanna			Univeristy of Michigan Health System	United States of America		
Rishi	Chanderraj			Univeristy of Michigan Health System	United States of America		
Maria	Pliakas			Univeristy of Michigan Health System	United States of America		
Ann	Wolski			Univeristy of Michigan Health System	United States of America		
Jennifer	Cirino			Univeristy of Michigan Health System	United States of America		
Dima	Dandachi			University of Missouri	United States of America		
Hariharan	Regunath			University of Missouri	United States of America		
Maraya N	Camazine			University of Missouri	United States of America		
Grant E	Geiger			University of Missouri	United States of America		
Abdoulie O	Njai			University of Missouri	United States of America		
Baraa M	Saad			University of Missouri	United States of America		
Faraaz Ali	Shah			University of Pittsburgh	United States of America		
Byron	Chuan			University of Pittsburgh	United States of America		
Sagar L	Rawal			University of Pittsburgh	United States of America		
Manal	Piracha			University of Pittsburgh	United States of America		
Joseph E	Tonna			University of Utah Health	United States of America		
Nicholas M	Levin			University of Utah Health	United States of America		
Kayte	Suslavich			University of Utah Health	United States of America		
Rachel	Tsolinas			University of Utah Health	United States of America		
Zachary T	Fica			University of Utah Health	United States of America		
Chloe R	Skidmore			University of Utah Health	United States of America		
Renee D	Stapleton			University of Vermont Larner College	United States of America		
Anne E	Dixon			University of Vermont Larner College	United States of America		
Olivia	Johnson			University of Vermont Larner College	United States of America		
Sara S	Ardren			University of Vermont Larner College	United States of America		
Stephanie	Burns			University of Vermont Larner College	United States of America		

Supplemental Online Content: Nonauthor Collaborators

*Indicates required information. Only first name, last name, and suffix will appear in PubMed.

*First Name and Middle Initial(s)	*Last Name	*Suffix (eg, Jr, III)	Academic Degrees	Institution	Location (city, state/province, country)	Role or Contribution, eg, chair, principal investigator	Group (if more than 1 Group listed in the byline) and/or Subgroup (eg, Steering Committee)
Anna	Raymond			University of Vermont Larner College	United States of America		
Erika	Gonyaw			University of Vermont Larner College	United States of America		
Kevin	Hodgdon			University of Vermont Larner College	United States of America		
Chole	Houseger			University of Vermont Larner College	United States of America		
Benjamin	Lin			University of Vermont Larner College	United States of America		
Karen	McQuesten			University of Vermont Larner College	United States of America		
Heidi	Pecott- Grimm			University of Vermont Larner College	United States of America		
Julie	Sweet			University of Vermont Larner College	United States of America		
Sebastian	Ventrone			University of Vermont Larner College	United States of America		
Nita	Khandelwal			University of Washington	United States of America		
T Eoin	West			University of Washington	United States of America		
Ellen S	Caldwell			University of Washington	United States of America		
Lara	Lovelace-Macon			University of Washington	United States of America		
Navya	Garimella			University of Washington	United States of America		
Denisse B	Dow			University of Washington	United States of America		
Murtaza	Akhter			Valleywise Health (formerly Maricopa	United States of America		
Rania Abdul	Rahman			Valleywise Health (formerly Maricopa	United States of America		
Mary	Mulrow			Valleywise Health (formerly Maricopa	United States of America		
Erin M	Wilfong			Vanderbilt University Medical Center	United States of America		
Kelsi	Vela			Vanderbilt University Medical Center	United States of America		
Ashish K	Khanna			Wake Forest University School of Me	United States of America		
Lynne	Harris			Wake Forest University School of Me	United States of America		
Bruce	Cusson			Wake Forest University School of Me	United States of America		
Jacob	Fowler			Wake Forest University School of Me	United States of America		
David	Vaneenenaam			Wake Forest University School of Me	United States of America		
Glen	Mckinney			Wake Forest University School of Me	United States of America		
Imoh	Udoh			Wake Forest University School of Me	United States of America		
Kathleen	Johnson			Wake Forest University School of Me	United States of America		
Patrick G	Lyons			Washington University School of Med	United States of America		
Andrew P	Michelson			Washington University School of Med	United States of America		

Supplemental Online Content: Nonauthor Collaborators

*Indicates required information. Only first name, last name, and suffix will appear in PubMed.

*First Name and Middle Initial(s)	*Last Name	*Suffix (eg, Jr, III)	Academic Degrees	Institution	Location (city, state/province, country)	Role or Contribution, eg, chair, principal investigator	Group (if more than 1 Group listed in the byline) and/or Subgroup (eg, Steering Committee)
Sara S	Haulf			Washington University School of Med	United States of America		
Lauren M	Lynch			Washington University School of Med	United States of America		
Nguyet M	Nguyen			Washington University School of Med	United States of America		
Aaron	Steinbery			Washington University School of Med	United States of America		
Nicholas	Braus			William S. Middleton Memorial VA He	United States of America		
Vishwanath	Pattan			Wyoming Medical Center	United States of America		
Jessica	Papke			Wyoming Medical Center	United States of America		
Ismail	Jimada			Wyoming Medical Center	United States of America		
Nida	Mhid			Wyoming Medical Center	United States of America		
Samuel	Chakola			Wyoming Medical Center	United States of America		
Kevin	Sheth			Yale New Haven Health New Haven	United States of America		
Abdalla	Ammar			Yale New Haven Health New Haven	United States of America		
Mahmoud	Ammar			Yale New Haven Health New Haven	United States of America		
Victor Torres	Lopez			Yale New Haven Health New Haven	United States of America		
Charles	Dela Cruz			Yale New Haven Health New Haven	United States of America		
Akhil	Khosla			Yale New Haven Health New Haven	United States of America		
Samir	Gautam			Yale New Haven Health New Haven	United States of America		
